# In vitro models to mimic tumor endothelial cell-mediated immune cell reprogramming in lung adenocarcinoma

**DOI:** 10.1186/s13046-025-03576-4

**Published:** 2025-11-27

**Authors:** Morgane Krejbich, Emilie Navarro, Judith Fresquet, Marine Cotinat, Valentin Isen, Hortense Perdrieau, Virginie Forest, Aurélie Doméné, Tiphaine Delaunay, Hala Awada, Vincent Dochez, David Roulois, Nicolas Boisgerault, Richard Redon, Christophe Blanquart, Isabelle Corre, Lucas Treps

**Affiliations:** 1 INSERM UMR 1307, CNRS UMR 6075, Nantes Université, Université d’Angers, Nantes, F-44000 France; 2 Honeycomb team, Univ Rennes, UMR S1236, Rennes, France; 3 CNRS, Inserm, l’institut du thorax, Nantes Université, CHU Nantes, Nantes, F-44000 France; 4 CNRS, Inserm, BioCore, US16, SFR Bonamy, Nantes Université, CHU Nantes, Nantes, F-44000 France; 5 Service de Gynécologie-Obstétrique, INSERM, CIC 1413, Nantes Université, CHU Nantes, Nantes, F-44000 France; 6 Movement - Interactions - Performance, MIP, EA 4334, Nantes Université, CHU Nantes, Nantes, F-44000 France

**Keywords:** Tumor endothelial cells, NSCLC, Tumor microenvironment, Immunity, Macrophages, Cancer-associated fibroblast, CAF, ScRNA-seq, Spheroid, 3D models

## Abstract

Tumor endothelial cells (TECs) play a critical role in regulating immune responses within the tumor microenvironment (TME). However, the mechanisms by which TECs modulate immune cell population remain unclear, particularly in non-small cell lung cancer (NSCLC). Here, we investigated how NSCLC cells tweak normal endothelial cells (NECs) into TECs and the subsequent effects on immune regulation. NECs were cocultured with various NSCLC cell lines, using 2D and 3D coculture models to evaluate TEC-mediated effects on immune cells. We show that direct coculture led to significant transcriptomic, proteomic and kinomic alterations in TECs, especially in pro-inflammatory pathways. We identified a downregulation of the co-stimulatory molecule OX40L in TECs compared to NECs, suggesting impaired T-cell proliferation support. While TECs showed a limited effect on CD8^+^ T-cell activation, TECs supported CD4^+^ T-cells polarization into Treg and Th22 subsets. Moreover, TECs also promoted M2-like macrophages polarization, thereby potentially contributing to the TME immunosuppression. State-of-the-art single-cell RNA sequencing of 3D multicellular tumor spheroids (MCTS) revealed formidable heterogeneity in the tumor cells and cancer-associated fibroblast compartments. It also unveiled distinct TEC subpopulations, including an inflammatory subset with an unfolded-protein response signature. This TEC cluster was absent in 2D-cultured NECs but present in freshly isolated and 2D-cultured TECs from NSCLC patients. Importantly, we identified a perivascular M2-like macrophage subset within MCTS that is in close contact with TECs, and is predicted to interact with them through MIF signaling. In conclusion, TECs in NSCLC tumors play a pivotal role in remodeling the TME immune landscape by promoting immune suppression. This study highlights the complex immunoregulatory functions of TECs within different in vitro models that mimic aspects of the TME. Our data may provide new insights into potential therapeutic strategies targeting TECs or regulatory signaling to improve the efficacy of immunotherapy in NSCLC.

**Supplementary Information:**

The online version contains supplementary material available at 10.1186/s13046-025-03576-4.

## Introduction

Responsible for an estimated 1.8 million deaths per year, lung cancer is one of the leading causes of cancer incidence and mortality worldwide, with non-small-cell lung cancers (NSCLC) accounting for 80–85% of cases [1]. Over the past decade, a better understanding of the biology of lung cancer, particularly the composition and activity of the tumor microenvironment (TME), has led to the development of new therapies that have improved survival [2]. As such, immunotherapy in the form of immune checkpoint inhibitors has revolutionized patient outcome compared to chemotherapy alone [3–6].

These immunotherapies are based on reversing the T lymphocyte anergic state induced by tumor cells and heterogeneous immune cells from the TME, including tumor-associated macrophages (TAM), regulatory T cells (Treg) and Th2/22 cells. However, tumor progression and response to treatment may also be affected by cells that support the architecture of the TME, including cancer-associated fibroblasts (CAFs), endothelial cells (ECs), and other factors such as the extracellular matrix. There is considerable evidence that tumor endothelial cells (TECs) have unique phenotypic and functional traits in comparison to normal endothelial cells (NECs) in terms of metabolism, genetics and transcriptomic profile [7]. Moreover, emerging evidence indicates that TECs display immunoregulatory features that could have therapeutic relevance for cancer patients and their response to immunotherapies [8–13]. Current assumptions are that some TEC subsets, in the form of tumor-associated high endothelial venules, are gatekeepers for TME infiltrating immune cells and essential for successful antitumor immunity [14], whereas other TECs act as semi-professional antigen presenting cells (APC) to activate or inhibit effector cells [15] and model tertiary lymphoid structures in the tumor [16]. The difficulty in studying TECs holds in the fact that they are highly heterogeneous and plastic by nature and because of the lack of relevant models to study their immunoregulatory properties. In this study we established various models to unravel how tumor cells could rewire NECs into TECs, and the impact of this remodeling on the immune system. Various approaches including single-cell RNA sequencing (scRNA-seq) and functional assays are employed to reach this aim.

## Materials & methods

### HUVEC isolation

Human umbilical vein endothelial cells (HUVECs) were freshly isolated from umbilical cords obtained from multiple donors from the CHU Maternity, Nantes. Informed consent was obtained from all subjects. The blood from umbilical cord is rinsed with PBS and sterile gauze and 10 ml of the 0.2% collagenase type I in 0.9% NaCl + 2 mM CaCl2 + 2X P/S (Penicillin/Streptomycin), PBS and M199 solution is added in the vein and the up side clamped with a sterile clip. The umbilical cord is incubated 13 min at 37 °C. After incubation, the umbilical cords are unclipped and the collagenase collected. The vein is rinsed with M199 medium and the cells are centrifuged at 300 x g for 5 min. The cells are resuspended into a M199 medium with 2X P/S and grown on a gelatin coated T75 flask in a 37 °C incubator with 5% of CO2.

### Cell culture

HUVECs were cultured in Endothelial Cell Growth Medium 2 (EGM2) media (22011B, Promocell) supplemented with 2% fetal calf serum (FCS), 5 ng/ml epidermal growth factor (EGF), 10 ng/ml basic fibroblast growth factor, 20 ng/ml Insulin-like Growth Factor (Long R3 IGF), 0.5 ng/ml VEGF 165, 1 µg/ml ascorbic acid, 22.3 µg/ml heparin, and 0.2 µg/ml hydrocortisone from the Supplement Mix (C-39216) in flasks coated with 0.1% gelatin (Sigma; #SLCF9893). Primary human lung microvascular endothelial cells (HMVEC-L; Lonza) were grown as described previously in EBM-2 endothelial basal medium with 5% FBS, EGM-2MV supplements (Lonza) [17]. ECs were used between passages 2–7. Healthy lung fibroblasts were isolated from patient biopsies (BREATHE clinical cohort - NCT0681813) and cultured in DMEM supplemented with 10% FBS. NSCLC cell lines, A549, H1755 and H1975 were cultured in RPMI 1640 medium (Gibco, Invitrogen) supplemented with 100 U/ml penicillin, 100 mg/ml streptomycin, 2 mM L-glutamine (Gibco, Invitrogen), 10% FBS (Corning), and cultured at 37 °C in a 5% CO_2_ atmosphere. All cells were tested each week to prevent mycoplasma contaminations using PlasmoTest™ (Invivogen), besides NSCLC cell lines were validated by STR to prevent derivation and cell cross contamination over passages.

### Monocyte and lymphocyte T-Cell purification

Peripheral Blood Mononuclear cells (PBMCs) were collected from healthy donors (Etablissement Français du Sang, ethics agreement CPDL-PLER-2022 09) and isolated using Ficoll gradient (Eurobio, Cat#CMSMSL01-01). Polyclonal CD8^+^ T cells were selected using the EasySep Human CD8^+^ T Cell Isolation Kit (STEMCELL Technologies; 17953), naive-activated CD4^+^ T cells were isolated using the EasySep Human naive CD4^+^ T Cell Isolation Kit (STEMCELL Technologies; 19555), and polyclonal CD4^+^ T cells were isolated using the EasySep Human polyclonal CD4^+^ T Cell Isolation Kit (STEMCELL Technologies; 17952). Blood monocytes were isolated through negative magnetic sorting using the EasySep Human Monocyte Enrichment Kit without CD16 depletion (STEMCELL Technologies; 19058). All of the aforementioned immune cells were isolated according to the manufacturer’s recommendations.

### Myeloid cell differentiation

Human monocytes were seeded in a 6-well plate at a concentration of 1 million/ml in full EGM2 media. As a first step, 100 ng/ml of M-CSF was added for 7 days to induce M0 macrophages. Thereafter, M0 macrophages were treated for 48 h with 2ng/ml of IFNγ and 50 ng/ml of LPS, or 10 ng/ml of IL-4 to induce M1- and M2-like macrophages, respectively [18].

### Coculture experiments

#### Coculture between HUVECs and NSCLC cell lines

We conducted our coculture according to the protocol suggested by Njock and his team [19]. The day before coculture HUVECs and NSCLC cell lines were rinsed with D-PBS and placed in EGM2 without growth factor (EGM2 SF) but completed with 0.5% FBS depleted of extracellular vesicles by ultracentrifugation and 5 ng/ml human Fibroblast Growth Factor (bFGF) (HY-P7004, MedChemExpress) overnight. The next day, HUVECs were seeded in EGM2 for 6 h, then tumor cells were added to plates with adherent HUVECs in a 1:1 ratio in EGM2 SF, 0.5% FBS exofree and 5 ng/ml human bFGF. After 48 h, monoculture (HUVECs alone) or coculture (HUVECs + A549/HUVECs + H1755/HUVECs + H1975) are detached with TrypLE Select (1X) (Gibco^TM^,12563029) and HUVECs were positively selected for CD31 by magnetic beads cell sorting (MACS, Miltenyi Biotec, 130-091−935) using LS separation columns (Miltenyi Biotec, 130-042−401) following the manufacturer’s instructions. Cells were passed sequentially over two separation columns to reach higher purity. Fully supplemented EGM2 was used for the final elution step from the column.

#### Coculture between NECs/NSCLC-TECs and T lymphocytes

Cocultures were performed between NECs or NSCLC-TECs with polyclonal CD4^+^, naive CD4^+^ or CD8^+^ T cells. After isolation, lymphocytes were activated using Dynabeads Human T-Activator CD3/CD28 (Thermo Fisher Scientific) with a ratio of 1 bead for 2 cells. The cells were placed in contact for 5 days in 2/3 fully supplemented EGM2 and 1/3 RPMI 1640 medium supplemented with 8% UltraGRO (AventaCell, Atlanta, GA), 100 U/mL penicillin, 100 mg/mL streptomycin, 2 mM L-glutamine, and 50 U/mL interleukin-2 (IL-2) (Proleukin, Novartis, Basel, Switzerland).

#### Coculture between NECs/NSCLC-TECs and monocytes

Cocultures were done between NECs or NSCLC-TECs with monocytes for 5 days in fully supplemented EGM2 media with a ratio of 1 NEC or TEC for 5 monocytes.

#### Triculture between A549, HUVECs and monocytes (AHM triculture)

Cocultures were done between HUVECs, A549 NSCLC cell line and monocytes for 5 days in fully supplemented EGM2 media with different cell ratios to study increasing amounts of ECs during the triculture: A549, HUVECs and monocytes (1:1:10 and 1:2:10). Milatuzumab treatment (10 µg/ml) was added in some of the experiments in both control and cocultures conditions during seeding before macrophages polarization.

### RNA isolation

After CD31 sorting, total RNA from HUVECs was extracted using the miRNeasy Micro Kit (Qiagen; 217084) according to the manufacturer’s instruction, and RNA integrity and profiles were measured before sequencing using the Agilent 2100 Bioanalyzer (Agilent) for total RNA (RNA nanochips).

### Reverse-transcriptase quantitative polymerase chain reaction (RT-qPCR)

One microgram of RNA was reverse transcribed using Revert Aid H Minus Reverse Transcriptase (ThermoFischer Scientific), and the RT product was used for expression analysis using Maxima SYBR Green/ROX qPCR Master Mix (ThermoFischer Scientific) on a QuantStudio3 system (Applied). Targets of interest were amplified using premade primer: *PECAM1* (QT00081172), *ICAM1* (QT00074900), *VCAM1* (QT00018347), *CD274* (PD-L1) (QT00082775), *TNFSF4* (OX40L) (QT00028658), *CD163* (QT00074641), *LYVE1* (QT00034566), *FOLR2* (QT00062608) and *HLA-DRA* (Forward: 5’CCTGTCACCACAGGAGTGTC; Reverse: 5’TCCACCCTGCAGTCGTAAAC). Gene expression was normalized based on the expression of the housekeeping gene encoding the Ribosomal Protein Lateral Stalk Subunit P0 (*RPLP0*).

### 3’RNA-seq digital gene expression and analysis

Gene analysis was performed thanks to the GenoBird platform by 3’RNA-sequencing profiling using a NovaSeq 6000 (Illumina). The quality of raw sequence reads was assessed by FastQC. Adapter sequences were trimmed off the raw sequence reads using Cutadapt. Reads were aligned to the human (hg38) genome using BWA. All obtained data have been uploaded on GEO Omnibus site (GSE247526). Bioinformatic analysis to compare gene expression signature and gene set enrichment analysis was carried out using the DESeq2 and pathfindR (min_gset_size = 10, max_gset_size = 300) packages. An adjusted *p*-value < 0.05 was considered statistically significant. A *meta-analysis* for commonly upregulated gene sets between HUVEC monocultures and NSCLC or MDA-MB-231 cocultures [19] was performed using the BIOMEX software with a product-rank approach [20]. *P*-values form the depicted gene set of the meta-analysis were calculated by Fisher exact test. *Survival analysis* was performed on lung cancer TCGA with the online tool Kaplan-Meier Plotter [21] using the following parameter: mean expression of selected genes, adenocarcinoma histology, all stage, split patients by median, univariate Cox regression.

### MCTS formation and scRNA-seq

MCTS were formed by mixing A549, normal primary HUVECs, normal primary human lung fibroblasts, and PBMC-derived monocytes at a defined ratio of 2:1:1:1, with a total of 20,000 cells per MCTS. Cells were mixed and loaded in a low adherence plate (Nunclon sphera, Thermofisher Scientific), and pelleted by centrifugation. Fully supplemented EGM2 medium was used to form and culture the MCTS. After 5 days of culture, MCTS were collected, rinsed with PBS and dissociated with TrypLE. The single cell suspension was then resuspended in a 0.04% PBS BSA and the viability assessed (> 85%). 33,000 cells were used to generate single cell droplet libraries with Chromium NEXT GEM Single Cell 3’ Reagent kit v3.1, 10X Chromium Single Cell Controller (10X Genomics, Pleasanton, CA, USA). Library size was determined by Agilent TapeStation assays and the concentration by Qubit Flex (Invitrogen). Libraries were pooled and sequenced on NovaSeq 6000 Sequencing System (Illumina), providing a read depth of > 20,000 read pairs per cell according to manufacturer’s instructions. A number of 4 samples was sequenced, representing different donors of fibroblasts, HUVECs and PBMC-derived monocytes. A549 cell line was the same across all 4 samples and used with no more than 2 passages in between the 2 batches of scRNA-seq.

### scRNA-seq bioinformatic analysis

Gene expression matrices were generated using Cell Ranger (10X Genomics, version 2.1.1) using the GRCh38 build of the human reference genome, and further processed using RStudio (version 2023.12.1 + 402). We used the following quality control steps: genes expressed by < 3 cells were not considered, cells expressing < 200 genes (low quality) or >10,000 genes, < 200 genes or >10,000 unique molecular identifiers (UMIs), or >12% of UMIs derived from the mitochondrial genome were removed. 279 doublets were detected and removed using the DoubletFinder package. Doublets represent ~ 0.5% of filtered cells and did not form any specific cluster nor were overrepresented in a particular sample. Visualization, clustering, marker gene identification and gene set enrichment analysis of the remaining cell data was performed using the SeuratV5 package. Since enzymatic and mechanical dissociations were described as eventually inducing a specific transcriptomic response, we checked if a specific cell cluster/subcluster was enriched for the dissociation signature [22]. Prior to *SCENIC analysis*, gene expression matrix was exported to a python compatible file using ScopeLoomR. Then, after 10 iterations of pySCENIC pipeline, results were aggregated in a custom python pipeline. Using only matrices resulting from the aggregation, the integration of regulons data was analyzed in Seurat and R (more details in the Supplementary methods). *Copy number variations* (CNV) were estimated using the *inferCNV* R package (version 1.5.0). For this analysis, we first performed the analysis without reference populations to visualize chromosomal alterations across all cell types. After identifying cluster with similar CNV, and matching these results with cluster gene signatures, we run a second time inferCNV with the immune cell, ECs and fibroblasts as reference cells. Analysis was performed using the following parameters: cut-off 0.1, denoise = T, min_cells_per_gene = 10, HMM = F, cluster_by_groups = F. *Cluster similarities* across different scRNA-seq dataset was assessed using ClusterMap R package on the marker gene signatures (min.pct = 0.25, logfc.threshold = 0.25, edge_cutoff = 0.05). *Signature enrichment scores* were calculated using the UCell R package using the Mann-Whitney U statistic. *CellChat* was performed using default parameters [23] on the full CellChat database by predicting interactions between the endothelial and myeloid compartments. *Cell trajectory* analysis was performed using Monocle R package (release 3.21).

### Protein Tyrosine Kinase (PTK) and Serine Threonine Kinase (STK) Kinome assay

After NECs or NSCLC-TECs isolation, cell pellets were collected and lysed with a M-PER TM reagent (ThermoFischer Scientific, 78501) containing protease and phosphatase inhibitors (ThermoFischer Scientific, 87785). Lysates were dosed using BCA protein assay kit (ThermoFischer Scientific, 23225) to make sure that we had enough protein quantity for all our experimental conditions and 3.35 µg and 1 µg of protein samples were loaded on the PTK and STK PamChip arrays, respectively (PamGene). Everything was processed using the manufacturer’s recommendations and using the PamStation 12. The array allows the tracking of the phosphorylation of 196 peptides for the PTK and of 144 peptides for the STK, VSN normalization was performed and kinase activities were predicted, and further checked for quality filtering. The data were analyzed by PamGene.

### Secretome proteomic analysis

Digestions and LC-MS/MS analyses were performed at the Prot’ICO proteomics facility as described previously [24]. NSCLC cell lines were incubated 72 h in RPMI with 10% SVFd and 1% P/S. Supernatants were washed away with 1X PBS and replaced by RPMI without SVF and allowed to condition this new medium for 24 h. Cells were counted for subsequent normalization, and media (~ 6 mL) were collected and centrifuged 5 min at 300 x g before proceeding to mass spectrometry analysis (Supplementary Methods).

### Immunoblotting analysis

NECs or NSCLC-TECs were lysed with 100 µl of RIPA (Sigma-Aldrich) buffer 1X supplemented with protease inhibitor cocktail (Fast protease inhibitor, Sigma-Aldrich) and phosphatase inhibitors (Phos STOP, Roche). Cell lysates were centrifuged for 30 min at 800 x g at 4 °C to remove debris and the supernatants were stored at −80 °C. Proteins were quantified using Bradford assay (Interchim). 10 µg of proteins were loaded on a 4–20% Mini-PROTEAN TGX™ Precast Protein Gel (#4561093, Biorad) and then transferred onto a nitrocellulose membrane. After 1 h of saturation in 5% BSA, TBS-Tween 0,1%, the membranes were incubated with primary antibodies p65 Ser 536 (Cell Signaling Technology, 3033) (1/1000), stripped and then incubated with the antibody against the total form of p65 (Cell Signaling Technology, 8242) (1/1000) overnight at 4 °C. Proteins were incubated with a Goat anti-Rabbit (#11-001−003) (1/5000) secondary antibody (Jackson ImmunoResearch) for 1 h at room temperature and then revealed with the Immobile Western Chemiluminescent HRP Substrate (WBKLS0500, EMD Millipore). Data were analyzed with the Fusion FX device (Vilber).

### Spatial multi-phenotyping and analysis (Phenocycler instrument)

The PhenoCycler instrument (Akoya Biosciences, USA) performs iterative annealing and removal of fluorophore-conjugated oligo probes to primary antibody-conjugated complementary DNA barcodes. Antibody panel was constructed using 6 ready-to-use commercially available PhenoCycler antibodies (Akoya Biosciences). FFPE human lung was sectioned to a thickness of 5 μm and directly adhered onto poly-L-lysine (Sigma) coated 22 × 22 mm coverslips (Akoya Biosciences). Tissue coverslips were stored at 4 °C until staining. The sample was incubated for 20 min at 55 °C on a hot plate and tissue was cooled down before staining. Tissue section was deparaffinized and rehydrated by immersing the coverslip through the following solution for 5 min each: twice in OTTIX (Diapath), twice in 100% Ethanol (VWR Chemicals), once in 90%, 70%, 50%, 30% Ethanol and twice in ddH2O. Antigen retrieval was performed in a hot water bath at 100 °C in a 1X Citrate Buffer (Sigma) for 20 min. After cooling at room temperature, tissue section was briefly washed twice in ddH2O for 2 min. Following hydration (Akoya Biosciences) step, tissue section was blocked in Staining buffer (Akoya Biosciences) at room temperature for 20 min and stained with the 6 plex PhenoCycler antibody panel in a humidity chamber 3 h at room temperature. To ensure that the antibodies remain attached to the antigen during the multicycles PhenoCycler imaging, protocol post-staining protocol had 3 fixation steps. After incubation with antibodies, tissue section was washed twice and post-fixated with 1.6% PFA (Sigma) Storage buffer (Akoya Biosciences). for 10 min at room temperature. Tissue sections were briefly washed three times in 1X PBS buffer (VWR Life Science) and incubated in a cold methanol solution (Sigma) for 5 min. Following washing, tissue section was fixated in final fixative solution (Akoya Biosciences) for 20 min at room temperature. Finally, tissue section was washed three times in 1X PBS buffer and was stored in Storage Buffer at 4 °C before PhenoCycler imaging.

PhenoCycler imaging was performed with an Axio Observer (Zeiss) inverted microscope equipped with an ORCA Flash 4.0 LT camera (Hamamatsu). The microscope is coupled to the Colibri 7 LED light source (Zeiss) and fluorescence was detected using the monoband filter Set 112 HE LED (Zeiss). The PhenoCycler experimental run was managed by the instrument controller software (v1.30.0.12, Akoya Biosciences) integrating with the Zeiss microscope. Nuclear DAPI (Akoya Bioscience) staining was used to design manually tiled regions of interest at the 5x magnification (N-Achro 5x/0.15 M27, Zeiss). Automated multiplex imaging was performed using a Plan Apo 20x/0.8 M27 Air objective (Zeiss) with a 325 × 325 nm pixel size using software autofocus repeated every tile before acquiring a 11 plane-z-stack with a z-spacing of 1.5 μm. Data are acquired as 3D stacks by cycles of 4 markers (including a recurrent nuclear DAPI staining). After acquisition, raw files were exported using the CODEX Instrument Manager (CIM, Akoya Biosciences). Data are processed by computing the more in focus image from the stack to take into account the potential tissue unflatness, all cycles are registered together based on the DAPI staining, and the fluorescence signal is corrected by background fluorescence based on blank cycles and normalized in intensity [25]. Cell segmentation was performed by using the Stardist algorithm. After segmentation, measurement data are transformed in FCS files and analyzed with the CODEX MAV software.

### Flow cytometry

#### To characterize ECs after sorting

Cell suspensions were stained for 30 min at 4 °C with anti-CD31-AlexaFluor 488 (Biolegend, 303110), anti-CD274-PE (BD Biosciences, 557924), anti-CD252-PE (Biolegend, 326308), anti-CD54-APC-Fire 750 (Biolegend, 353122), anti-CD106-APC (Biolegend, 305810). Cells were washed twice with PBS, processed by flow cytometry on a 5 laser, 32 detector BD FACSymphony™ A5 Cell Analyzer (BD Biosciences) (Acquisition software: Diva 8). Median fluorescence intensities measured in flow cytometry experiments are normalized on the monoculture conditions and are displayed as ratios of median fluorescence intensities (RMFI) ± standard error of the mean (SEM).

#### To characterize macrophage populations cocultured with NECs/NSCLC-TECs

After washing in PBS, the cells were incubated 20 min with 1:500 Zombie UV™ Fixable Viability dye (Biolegend) in PBS. They were washed with PBS-0.1% Bovine Serum Albumine (BSA, Sigma-Aldrich) and fixed with PBS-4% paraformaldehyde (PFA, Electron Microscopy Sciences) for 10 min, washed and stored at 4 °C. Cells were incubated in 50 µL of Brilliant Stain Buffer (BD Biosciences) supplemented with 2.5 µL of human BD Fc Block (BD Biosciences) and 2.5 µL of True Stain Monocytes Blocker (BioLegend). After 20 min, appropriate concentrations of specific antibodies were added for 30 min at 4 °C in the dark with 1 µg/ml anti-CD31-AlexaFluor 488 (Biolegend, 303110), 1:800 anti-CD45-BUV805 (BD Biosciences), 1:200 anti-CD163-BV711 (BD Biosciences), 1:400 anti-HLA-DR-PE Vio770 (Miltenyi), 1 µg/ml anti-CD16-BUV737 (BD Biosciences), 1:100 anti-CD14-APC Vio770 (Miltenyi), 1:50 anti PD-L1-BV421 (BioLegend, 329714), 1:200 anti-CD80-BUV 395 (BD Biosciences, 565210), 1:200 anti-CD86-APC (Miltenyi,130-116−161>) and 1:200 anti-CD206-BV786 (BD Biosciences, 740999). After three washes in PBS-0.1%BSA, stained cells were analyzed on a BD FACSymphony™ A5 Cell Analyzer. The BD FACSDiva 8.0 software (BD Biosciences) was used for data acquisition and analyzed with the FlowJo VX software.

### OmiQ analysis

tSNE, PCA, clusters identification and Heatmap were done with OMIQ 2025 Dotmatics using the functions Gating Metaclusters and FlowSOM functions on CD31^−^CD45^+^ cells from the triculture AHM conditions and the M1 and M2-like macrophages profile, with or without milatuzumab treatment.

### CFSE

Polyclonal/naive CD4^+^ T cells, and polyclonal CD8^+^ T cells were purified by Ficoll gradient from peripheral PBMCs of healthy volunteers and stained with CellTrace CFSE (ThermoFischer Scientific) at 1µM as per the manufacturer’s instructions. CFSE stained cells are then cocultured with NECs or NSCLC-TECs for 5 days as per the ‘Coculture experiments’ section described previously. Thereafter, cells from the coculture are harvested with TryPLE and stained before being analyzed by flow cytometry. After washing in PBS, the cells were incubated 20 min with 1:500 Zombie UV™ Fixable Viability dye (Biolegend) in PBS. They were washed with PBS-0.1% BSA and incubated in PBS-0.1% BSA with anti-CD25-BV421 (BD Biosciences, 564033) and anti-CD4-APC (BD Biosciences, 555349) for extracellular staining 30 min 4 °C in the dark, washed 2 times with PBS and fixed with PBS-4% PFA for 10 min, washed and stored at 4°C. After fixation, cells were stained for intracellular proteins with PBS 0.5% Triton with an anti-FoxP3 PE antibody (BD Biosciences, 560046) for 30 min 4 °C in the dark. After three washes in PBS-0.1%BSA, stained cells were acquired on the BD FACSymphony.

### Confocal microscopy

Cocultures were seeded into 24-well plates on coverslips for 48 h and then fixed in PBS-4% PFA during 20 min at room temperature (RT). PBS-2%Triton-1%BSA was used to permeabilize the cells for 30 min at RT. Primary antibodies anti-CD31-488 (Biolegend, 303110) anti-vWF (Abcam, ab6994), CD163-594 (Biolegend, 364304), CD45-647 (Biolegend, 304018) were incubated with the cells overnight at 4 °C, and secondary antibodies AlexaFluor 594 (Cell Signalling Technology, 8889 S) were incubated for 1 h at RT with Hoechst 33,342 (Sigma, 14533) and anti-Phalloidin-647 (Invitrogen, A30107). Pictures were taken with a Nikon A1rHD LFOV confocal microscope, with a 60x oil immersion objective (Nikon Instruments). The images were processed and analyzed using the Fiji software [26].

After formation, MCTS spheroids were collected in PBS and permeabilized in PBS Triton 2%. After primary and secondary antibody incubations, MCTS were rinsed and immobilized on a IBIDI 8p with Cell-Tak (Corning). Finally, MCTS are transparized using RapiClear (SunjinLab, Nikon) 1.47 before image acquisition.

### ELISA assay

Cytokine production was quantified from cocultured supernatants 24 h after exposing T cells to HUVECs. TNF-α (Invitrogen, 88–7346−88) and IL-10 (Biolegend, 430604) secretions were measured according to the manufacturer’s instructions ELISA kits. Absorbance values were read at 450/570nm using Multiskan FC microplate reader (ThermoFischer Scientific) and concentrations were calculated according to the manufacturer’s instructions.

### Chemokine detection by multiplex assay

The supernatants of cocultures between sorted HUVEC and naive CD4^+^ T Lymphocytes were collected after 5 days. Cytokines such as IL-5, IL-13, IL-2, IL-6, IL-9, IL-10, IFNγ, TNF-α (TNFSF2), IL-17 A, IL-17 F, IL-4, IL-22 were quantified using the LEGENDplex™ Human T Helper Cytokine Panels Version 2 (BioLegend) according to the manufacturer’s recommendations.

### Leukocyte adhesion assay

After CD31 sorting, HUVECs were grown in fully supplemented EGM2 in 24-well plates coated with 0.1% gelatin to obtain a confluent cell monolayer during 6–24 h. Over this period, the activated monoculture control is exposed to a cytokine cocktail (6nM TNF-α and IL1-β). PBMCs were obtained as described above and labelled with calcein AM (Life, C3100MP). Medium was removed and HUVECs were washed with PBS before the addition of 2.0 × 10^6^ cells and incubation for 1 h at 37 °C. Non-adherent cells were removed by delicate washing 7 times with PBS. Cellular adhesion rates were determined by live cell imaging using the IncuCyte (Sartorius). Around ten fields-of-view per well are randomly chosen and analyzed with Fiji [26] to determine the number of adherent leukocytes per field of view.

### Leukocyte and monocyte chemotaxis

PBMCs isolated from the peripheral blood of healthy donors are placed in an 8 μm transwell insert and coculture media from monoculture of NECs or NSCLC-TECs is placed in the lower chamber. After 3 h of incubation, cells that have performed transmigration are collected, centrifuged, and transferred to a conical 96 well plate for staining with a PBS mix containing 1:100 anti-CD14-BV786 (BD Biosciences, 563698), 1:50 anti-CD3-BUV395 (BD Biosciences, 563546), 1:50 anti-CD4-BV510 (Biolegend, 317443) and 1:400 anti-CD8-PeCy7 antibodies (BD Biosciences, 557748). After staining 30 min in the dark at 4 °C, cells were washed and 100 µL of counting beads (Invitrogen 01–1234−42) were added before proceeding to flow cytometry analysis.

### Spontaneous EC migration assay

After CD31^+^ sorting, a 96-well plate was coated with gelatin 0.1%, and 5.10^3^ NECs or NSCLC-TECs were seeded per well. Thereafter, the plate was monitored in the Incucyte S3 (Sartorius) for live imaging. Phase images were acquired for every experiment. Five images of each well were taken every hour during 24 h in order to be able to track the cells in a movie. On Fiji [26], using the plugins « Tracking » with « Manual Tracking », these tracking parameters were selected: time interval: 60 min, x/y calibration: 1.24 μm, z calibration: 0. Two cells per image are tracked for 24 h.

### Scratch wound healing assay

Confluent HUVEC monolayers were pretreated for 24 h with 1 µg/ml Mitomycin C to block proliferation. A scratch wound was made using a 200 µl tip. After scratch, cells were washed with PBS and then incubated in supplemented EGM2 in the Incucyte incubator. Photographs were taken every 3 h for 15 h. Migration was measured with Fiji [26] and expressed as percentage of wound closure (gap area at T0 minus gap area at T6 in percentage of gap area at T0).

### Quantification and statistical analysis

Data represent mean ± SEM between biological replicates. Statistical significance was calculated using non-parametric Wilcoxon test or Kruskal-Wallis depending on the comparison. *p < 0.05; **p < 0.01; ***< p0.001. Statistical analyses were conducted using GraphPad PRISM.

## Results

### Coculture with NSCLC cells lead to profound transcriptomic EC alterations

**Fig. 1:**
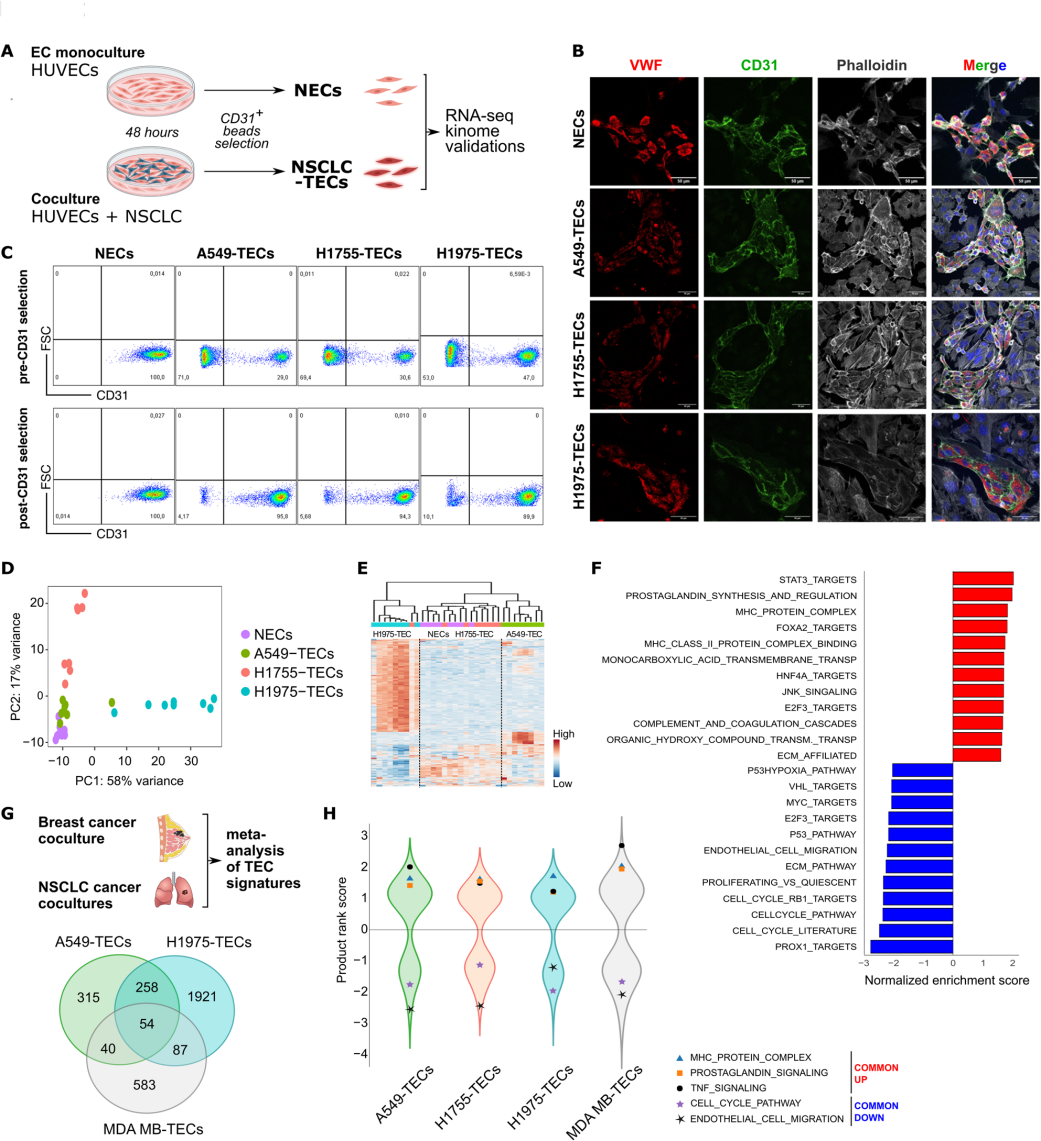
Coculture with Non-small Cell Lung Cancer (NSCLC) cells lead to profound transcriptomic endothelial cell alterations. **A**) Schematic of the 2D *in vitro* coculture system where human endothelial cells (ECs; HUVECs) were cocultured with/without NSCLC cell lines during 48h. Thereafter, normal (NECs) and NSCLC-cocultured ECs (NSCLC-TECs) were isolated using anti-CD31 antibody-coated magnetic beads, and 3’RNA-seq, kinome and functional validations were performed. **B**) Immunostaining for EC-specific markers (vWF in red and CD31 in green) revealed the spatial organization of HUVECs in cocultures. Scale bar: 50 μm. **C**) Flow cytometric analysis of different cocultured cells stained for CD31 before (i) and after (ii) CD31-enrichment. **D**) Principal component analysis considering the top 100 highly-variable genes. Samples segregate into 3 major groups with H1755-TECs being more similar to NECs as compared to the other NSCLC-TECs. *n*=8 per group. **E**) Correlation heatmap of the top 350 highly variable genes in NECs/NSCLC-TECs.** F**) Gene set enrichment analysis in all NSCLC-TECs *versus* NECs. Pathways enriched or downregulated in NSCLC-TECs appear in red and blue, respectively. Adjusted *p*-value < 0.05. **G**-**H**) Meta-analysis between NSCLC-TECs and HUVECs cocultured similarly with the breast cancer MDA-MB-231 cell line. Results show congruent up/downregulated targets at the **G**) gene and **H**) gene set levels

To unravel how cancer cells shape on normal endothelial cells (NECs) behavior we adapted a recent model of 2D coculture [19]. For this purpose, we selected three NSCLC cell lines (namely A549, H1755, H1975) that encompass the mutational diversity and aggressiveness traits of NSCLC (Fig. 1A; Supplementary Table S1) and cocultured them in direct contact with a well-established primary cultured model of normal ECs (HUVECs: human umbilical vein ECs) (Fig. 1A). Hereafter, HUVEC monoculture is referred to as control normal ECs (NECs), and NSCLC cell-cocultured HUVECs as tumor-like ECs (A549-TECs, H1755-TECs, H1975-TECs). Immunostaining for endothelial markers (vWF and CD31) revealed the spatial organization of HUVEC cocultures as surrounded by tumor cells (Fig. 1B). After 48 h of contact with NSCLC cells, NECs/TECs were enriched using magnetic beads coupled with CD31 antibodies. As *PECAM1* (the gene encoding for CD31), was not detected in NSCLC by real-time qPCR analysis (Supplementary Figure S1A), CD31 expression in coculture relies on ECs. Flow cytometry analysis of purified cells showed an enrichment for CD31, ranging from 89.9 to 95.8% of CD31^+^ cells (Fig. 1C), highlighting the endothelial purity of the CD31-purified fraction from HUVEC/NSCLC cocultures.

We then carried out an unbiased transcriptomic analysis to screen for global changes induced in NECs and TECs. Principal component (PCA) and hierarchical clustering analysis of the top-100 highly variable genes revealed that NECs and TECs grouped into 3 distinct clusters (Figs. 1D-E). Compared to H1975 and A549 that had a profound impact on the endothelial transcriptomic signature, H1755 minimally affected HUVECs with 3408, 667 and 41 significantly deregulated genes (adjusted *p*-value < 0.05), respectively (Supplementary Figure S1B; Supplementary Table S2). Interestingly, H1975- and A549-TECs had 335 commonly deregulated genes, and only 13 genes were in common between the 3 cocultures (Supplementary Figure S1C; Supplementary Table S2), suggesting that difference mediated by NSCLC/EC cocultures could be attributed to distinct genomic alteration profiles. Notably, the impact mediated by NSCLC onto trancriptomics of ECs seems to rely on direct cell-cell contacts as indirect coculture (by mean of inserts) did not show any significantly deregulated genes (adjusted *p*-value < 0.05) and PCA showed that most differences were mediated by the HUVEC donors and not by our experimental conditions (Supplementary Figure S1D-E). Gene set enrichment analysis (GSEA) was then performed to identify key upregulated pathways in TECs compared to NECs upon direct coculture. Among the 60 significantly upregulated pathways induced upon direct NSCLC cocultures, one third (21 gene sets) are associated with EC activation and pro-inflammatory signaling such as the prostaglandin synthesis, STAT3, TNF-α and complement pathways, IL-22 signaling, MHC class II proteins, and leukocyte cell adhesion (Fig. 1F). On the other hand, gene sets involved in cell cycle, proliferation, EC migration, pseudopodia formation and angiogenesis were downregulated. When comparing each H1975-, A549 or H1755-TECs to the NECs by GSEA we confirmed unbiased PCA and hierarchical clustering indicating a minimal impact of the H1755-NSCLC cell line on ECs (Supplementary Figure S1F). Thereafter, to maximize the relevance of our findings, we then performed a transcriptomic meta-analysis with RNA-sequencing dataset of HUVECs cocultured with the MDA MB-231 breast cancer cell line (hereafter coined as MDA MB-TECs [19]). Our meta-analysis revealed congruent deregulated gene signatures between MDA MB-TECs and NSCLC-TECs as compared to NECs. Indeed, gene sets related to MHC protein complex, prostaglandin signaling, cell-cell (leukocyte) adhesion and TNF signaling were upregulated, while cell proliferation and migration was again downregulated (Fig. 1G; Supplementary Tables S2-S3). Collectively, our data indicate that the pro-inflammatory phenotype acquired by ECs upon 2D direct coculture with tumors cells is probably not a unique feature of NSCLC but could also be generalized to other cancer types.

### Coculture with NSCLC cells altered EC proliferation and migration

We first investigated the proliferation defect of cocultured HUVECs (NSCLC-TECs) as suggested by GSEA. Immunostaining of CD31 with the proliferation marker KI67 indeed showed a much lower fraction of double positive CD31^+^KI67^+^ cells in cocultures compared to the monoculture condition (Supplementary Figure S2A). However, when NSCLC-TECs were purified and placed back in culture, no significant reduction in cell proliferation was observed, suggesting that the proliferation blockade could be partly mediated by contact inhibition in the NSCLC cocultures (Supplementary Figure S2B). Seemingly, the reduced migration profile in the coculture outlined by GSEA was not validated functionally in purified NECs/TECs that displayed similar migration and angiogenesis capacities in spontaneous, scratch wound and tubulogenesis assays (Supplementary Figures S2C-E). Altogether, our data suggest that the diminished proliferation and migration phenotype harbored by NSCLC-TECs during cocultures is rapidly lost when ECs are removed from this environment.

### Coculture with NSCLC cells lead to a pro-inflammatory and endothelial activation state

**Fig. 2:**
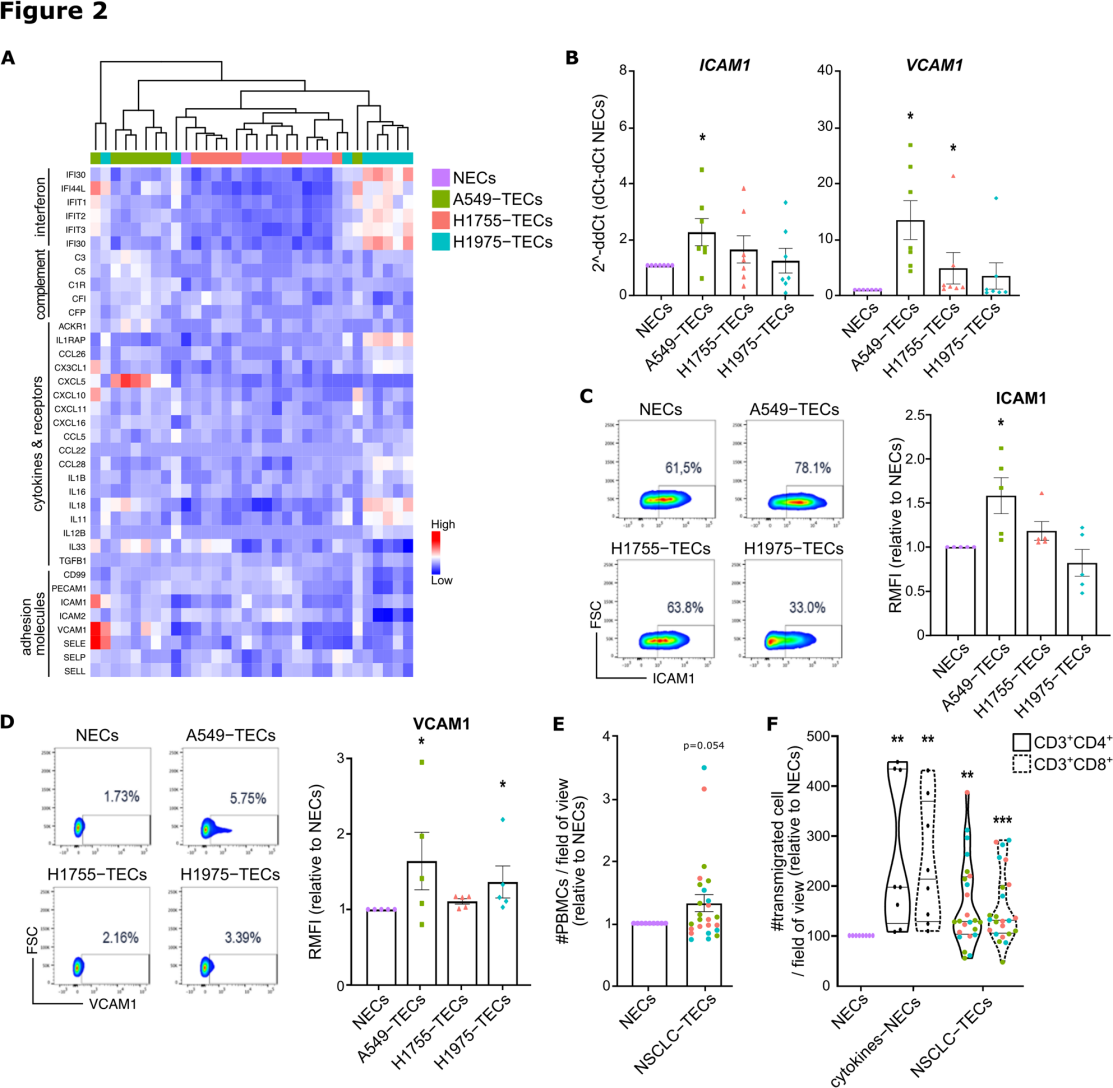
Coculture with NSCLC cells lead to a pro-inflammatory and endothelial activation state. **A**) Correlation heatmap for genes involved in pro-inflammatory pathways, cytokines and adhesion molecules required for leukocyte recruitment. Expression analysis for VCAM1 and ICAM1 by **B**) RT-qPCR and **C**-**D**) flow cytometry analysis in NECs/NSCLC-TECs. RMFI: Relative mean of fluorescence. **E**) NECs and NSCLC-TECs were cultured at confluency for 6 h after coculture and leukocyte adhesion assessed. **F**) Chemotaxis experiment for CD3^+^CD4^+^/CD8^+^ lymphocytes attracted by the coculture medium from cytokines-stimulated NECs or NSCLC-TECs. Data are mean ± SEM, n >5, *p < 0.05, **p < 0.01, Wilcoxon test compared to NECs

Differential expression analysis and GSEA revealed a marked pro-inflammatory and activation phenotype in all coculture conditions with the expression of interferon (*IFIT1-3*) or complement-related molecules (*C3*, *C5*, *C1R*), multiple cytokines and chemokines (*CCL26*, *CXCL10*,* IL16*, *IL18*), and adhesion molecules meant to attract and retain leukocytes (*ICAM1*, *VCAM1*,* SELE*) (Fig. 2A). We first confirmed by qPCR and flow cytometry the heightened expression of the two major (pro-inflammatory) adhesion molecules ICAM-1 and VCAM-1 in NSCLC-TECs (Figs. 2B-D). Notably, the induction of *ICAM1* and *VCAM1* in NSCLC-TECs appears relatively mild compared to NECs stimulated by the pro-inflammatory cytokines TNFα and IL-1ß (cytokines-NECs) which result in 40- and 150-fold changes, respectively (Supplementary Figure S3A). We hypothesized that increased expression of intercellular adhesion molecules by TECs could stimulate the attraction and binding of leukocytes onto the EC surface. Purified NSCLC-TECs were allowed to form a confluent monolayer for 6 and 24 h, and peripheral blood mononuclear cells (PBMCs) derived from healthy donors were isolated and placed onto NECs/TECs. Interestingly, while there was a trend to increase leukocyte adhesion at 6 h for NSCLC-TECs (and particularly for A549-TEC that expressed the higher level of ICAM-1 and VCAM-1) (Fig. 2E; Supplementary Figure S3B), this effect was lost after 24 h of culture (Supplementary Figure S3C). As TECs display an altered cytokine profile upon NSCLC coculture (Fig. 2A), we exposed PBMCs to NSCLC-TEC/NEC secretomes and measured immune cell chemotaxis with a transwell migration assay. Compared to the NEC secretome, we observed a trend towards increased transmigration for CD3^+^CD4^+^ and CD3^+^CD8^+^ cells for all NSCLC-TEC secretomes, with significant results for H1755- and H1975-TECs (Fig. 2F; Supplementary Figure S3D). There was no impact on the CD14^+^ myeloid chemotaxis using NSCLC-TEC secretomes (Supplementary Figure S3E).

### NSCLC-TECs have limited impact on CD8^+^ T cell activation

Having shown that NSCLC-TECs could promote CD3^+^ T cell recruitment, we sought to characterize their impact on lymphocyte activation and polarization. Indeed, in several tumor entities including NSCLC, the TME has been shown to induce CD8^+^ T cell exhaustion, which is the main target of immunotherapies [27]. After exposing HUVECs or not to NSCLC, NECs/TECs were subsequently cocultured with polyclonal activated CD8^+^ T cells for 24 h. In that condition, our flow cytometry analysis did not show significant variation of the late/middle T cell activation marker CD25 in CD3^+^CD8^+^ T cells, and only minimal changes in the secretion of TNFα usually produced by activated T cells, as measured by ELISA (Supplementary Figures S3F-G). Hence, although NSCLC-TEC secretomes increase CD8^+^ T cell attraction, TECs have limited impact on their activation in our direct 2D-coculture assay.

### NSCLC-TECs have reduced expression of co-stimulatory molecules

**Fig. 3:**
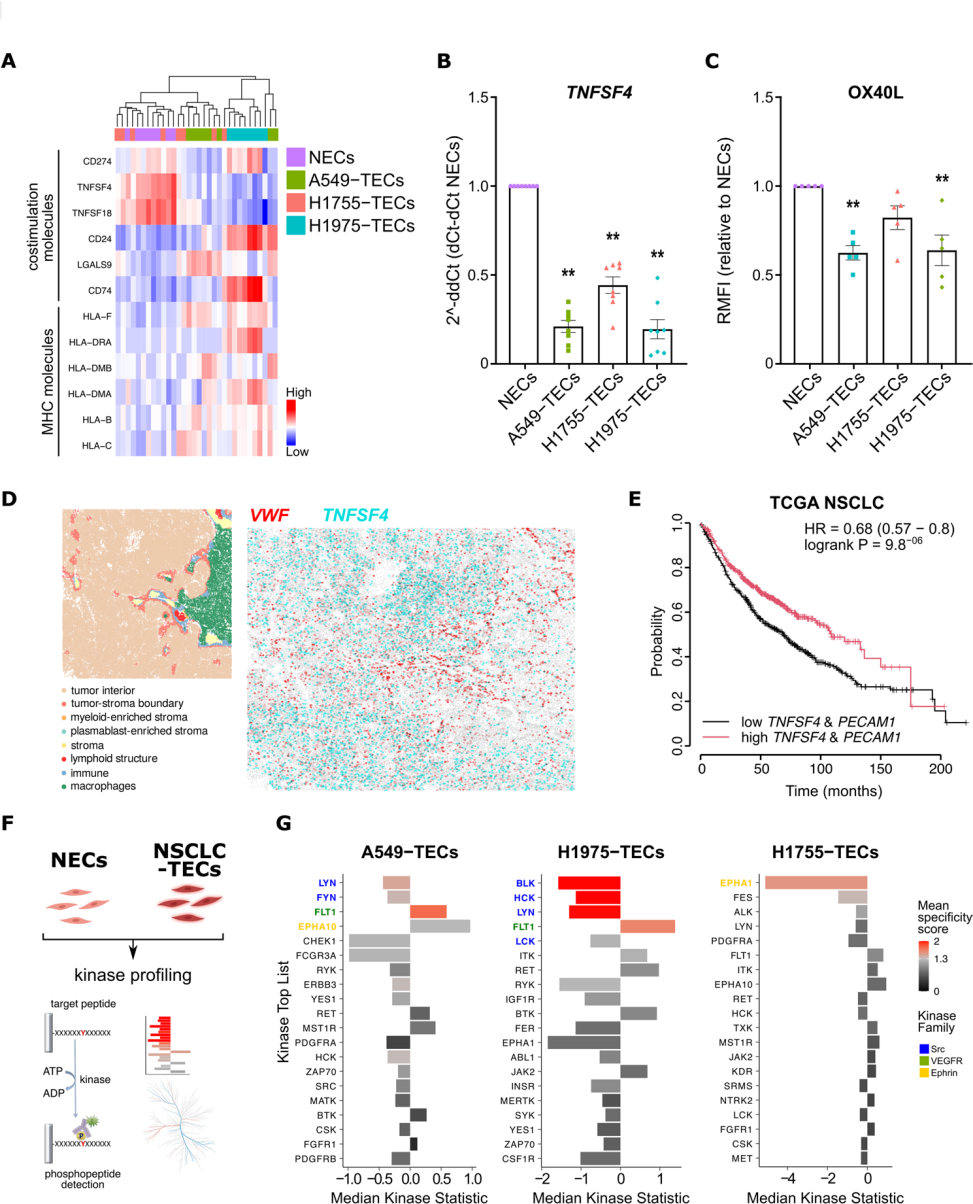
NSCLC-TECs have reduced expression of co-stimulatory molecules. **A**) Correlation heatmap analysis for genes involved in costimulation molecules and major histocompatibility complexes (MHC). Expression analysis for OX40-L (encoded by *TNFSF4*) by **B**) RT-qPCR and **C**) flow cytometry analysis in NEC/NSCLC-TECs. RMFI: Relative mean of fluorescence. **D**) Expression of *vWF* and *TNFSF4* in NSCLC biopsies dataset using CosMx spatial multiomics. Left inset shows the different niches determined in the biopsy. **E**) Correlation analysis between survival probability and co-expression of *TNFSF4* and *PECAM1* using TCGA NSCLC database. **F**) Schematic representation of kinase profiling in NECs/NSCLC-TECs. **G**) Score plot analysis of protein tyrosine kinases differentially regulated in NECs and NSCLC-TECs. A negative median kinase statistic means a downregulated kinase compared to NECs, and a specificity score in red is statistically significant. Kinase families are also represented in blue, green and yellow

Immunosuppression of the TME is also partly imposed by (but not limited to) pro-tumor CD4^+^ Treg and Th2 polarization [28]. This polarization can be achieved via various cytokines produced by cancer cells (e.g. TGFß), but also by direct contact with co-stimulatory molecules expressed on (semi)-professional APC such as ECs [29] (Fig. 3A). One of these molecules is OX40L (encoded by *TNFSF4*), and although it was previously described to be constitutively expressed by ECs [30] it has never been assessed yet in a context of cancer. We first confirmed by qPCR and flow cytometry the reduced expression of OX40L in NSCLC-TECs as compared to NECs (Figs. 3B-C). Of note, compared to other genes *TNFSF4* expression appeared relatively stable in HUVECs over 72 h after EC purification (data not shown). Interestingly, the reduced expression of OX40L in cocultured-HUVECs was also found in ECs from another vascular bed, namely lung microvascular EC (HMVEC-L), suggesting that this is not a unique feature of a single EC model (Supplementary Figure S4A). We then confirmed a tendency toward a reduced OX40L expression in CD45^−^CD31^+^ cells isolated from NSCLC patient biopsies as compared to healthy proximal and distal tissues. Interestingly, OX40L levels also appeared to be reduced in immune (CD45^+^CD31^−^) and vascular (CD45^−^CD31^+^) cells from malignant lung tissues (Supplementary Figure S4B). When exploring CosMx spatial multiomics NSCLC dataset, we noticed that *TNFSF4* was lowly expressed in vascularized stroma depicted by high vascular markers expression such as *VWF* (Fig. 3D). This reinforces our in vitro coculture data and suggest that OX40L is less expressed in vessels found in the tumor stroma. These findings have therapeutic relevance, as combined high *TNFSF4* and *PECAM1* expression (as associated with OX40L-expressing blood vessels in tumors) is associated with better overall survival in NSCLC patients (HR = 0.68; Fig. 3E). This was also tested for other endothelial markers and correlated with better overall survival in NSCLC patients (data not shown). In addition, high *TNFSF4* expression is associated with better overall survival probability (HR = 0.71) in cancer patients treated with immunotherapies (Supplementary Figure S4C). Indeed, OX40-OX40L interaction is described to (i) abolish the suppressive activity of FOXP3^+^ Tregs, (ii) prevent the induction of Tregs from effector T-cells, and (iii) induce the proliferation of memory and effector T lymphocytes [31]. OX40/OX40L thus appears as an important immunoregulatory signal, with OX40L expression tightly regulated by several actors including kinases of the PI3K, Src family in mast cell and dendritic cells [32, 33]. To unravel how the OX40L could be regulated in ECs, we performed a kinome assay to screen for 144 serine/threonine kinase (STK) and 196 protein tyrosine kinases (PTK) in TECs compared to NECs. After quality check filtering of the phosphorylated phosphosites and VSN normalization, an upstream kinase analysis algorithm was used to predict differential kinase activity in TECs compared to NECs (Fig. 3F). Mirroring the results obtained by RNA-sequencing (Figs. 1D-E), phylogenetic coral trees showed that A549- and H1975-TECs presented similar modifications in their kinome profile while H1755-TECs have limited effect size compared to NECs (Fig. 3G; Supplementary Figures 4D-E). Moreover, the majority of significantly deregulated predicted kinases appeared downregulated in A549- and H1975-TECs, with common kinases belonging to the Src kinase family (LYN, FYN, HCK, BLK) (Fig. 3G). In immune cells, LYN and FYN were previously reported to tightly control OX40-OX40L signaling, with the NFκB pathway directly implicated in this process [33–35]. As the NFκB transcripts *NFKB1* and *RELA* (encoding p50 and p65, respectively) were downregulated in H1975-TECs (Supplementary Figure 4F), and that the phosphorylated form of p65 (a mark for the NFKB canonical pathway activation) was diminished in H1975- and A549-TECs (Supplementary Figure 4G), our data suggest that the downregulation of LYN, FYN kinases and the NFκB signaling could explain the reduced OX40L expression in NSCLC-TECs.

### NSCLC-TECs mediate the polarization of pro-tumor CD4^+^ T cell subsets

**Fig. 4:**
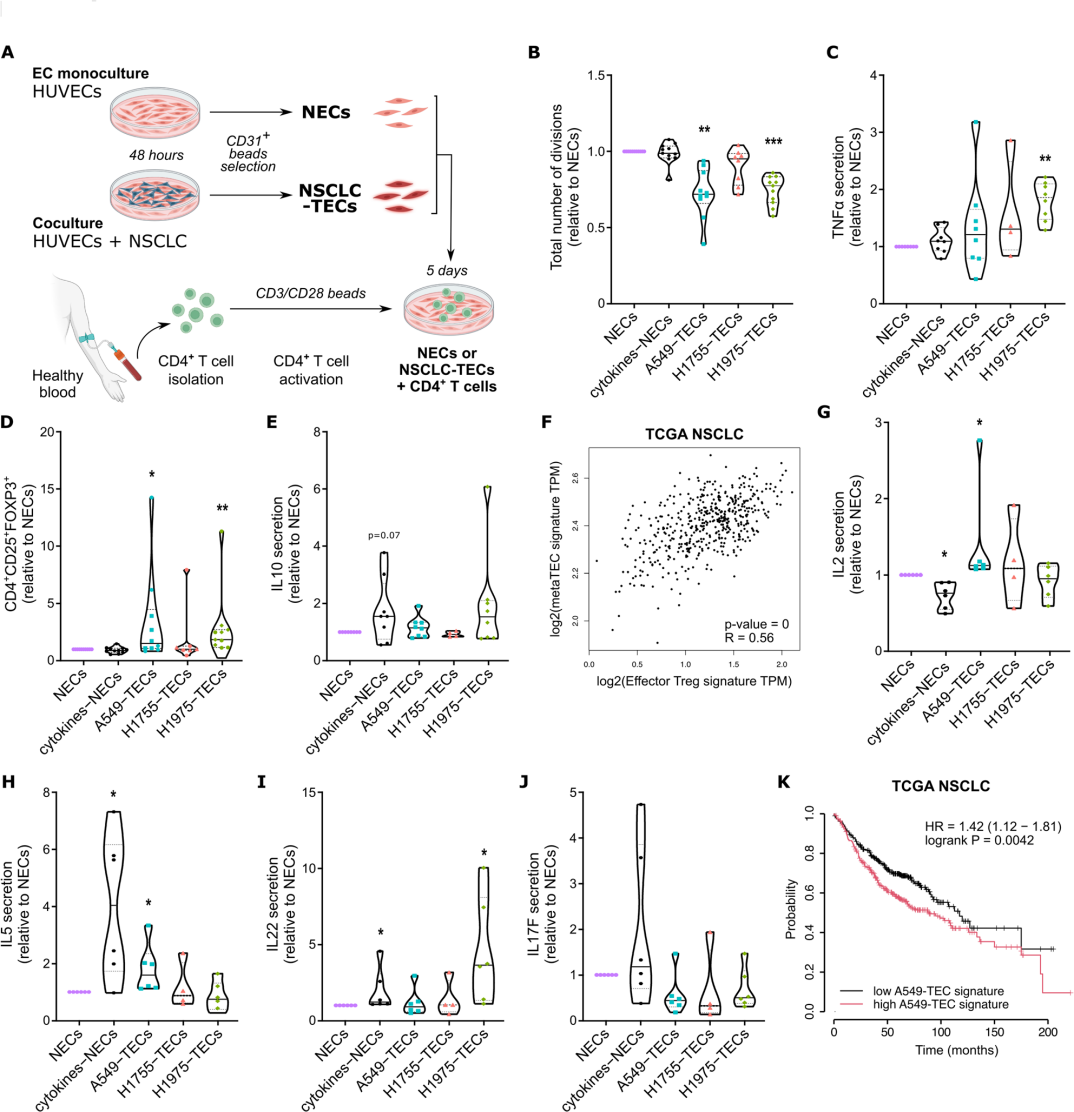
NSCLC TECs mediate the polarization of pro-tumor CD4^+^ T cell subsets. **A**) Schematic representation of the 5 days coculture between CD4^+^ T cells and NECs or NSCLC-TECs. **B**) Proliferation of polyclonal CD4^+^ T cells in cocultures with NECs/NSCLC-TECs and assessed with CFSE by flow cytometry. **C**) TNFa secretion dosed by ELISA in the coculture supernatant from polyclonal CD4^+^ T cells cocultured with NECs/NSCLC-TECs. **D**) Fraction of CD4^+^CD25^+^FOXP3^+^ Treg in the polyclonal CD4^+^ T cells/EC cocultures. **E**) IL10 secretion dosed by ELISA in the coculture supernatants from polyclonal CD4^+^ T cells with NECs/NSCLC-TECs. **F**) Correlation between our meta-analysis TEC signature (Figure 1G) and the effector Treg signature in NSCLC TCGA database. **G**) IL2, **H**) IL5, **I**) IL22 and **J**) IL17F cytokines secretion relative to NECs using LegendPlex in coculture supernatants from naive CD4^+^ T cells with NECs/NSCLC-TECs. **K**) Survival probability of NSCLC patients from the TCGA database regarding a low or high A549-TEC signature. Data are mean ± SEM, n>4, *p < 0.05, **p < 0.01, Wilcoxon test compared to NECs

Following the same strategy as for CD8^+^ T cells, we scrutinized the impact of NECs/TECs on polyclonal or naive-activated CD4^+^ T cells (Fig. 4A). When cocultured with NSCLC-TECs, flow cytometry analysis of CFSE-labelled polyclonal CD4^+^ T cells showed a significantly diminished proliferation with A549- and H1975-TECs (Fig. 4B). Conversely, naive CD4^+^ T cells proliferation was higher when cultured with H1755-TECs compared to NECs (Supplementary Figure S5A). The amount of TNF-α was quantified by ELISA in polyclonal CD4^+^ T cell/EC coculture and showed a 1.8-fold change elevation when H1975-TECs were used compared to NECs (Fig. 4C). Besides being secreted by activated T cells, TNF-α is described to act on Treg polarization. As such the fraction of CD4^+^CD25^+^FOXP3^+^ Treg was higher with A549- and H1975-TECs for polyclonal CD4^+^ T cells but not affected in naive CD4^+^ T cells (Fig. 4D; Supplementary Figure S5B). Seemingly, there was a tendency to an increased secretion of IL-10, a cytokine secreted by Treg, in cocultures with polyclonal CD4^+^ T cells and A549- or H1975-TECs (Fig. 4E). Further highlighting the clinical relevance of our findings, the TEC signature obtained from our meta-analysis (Fig. 1G; Supplementary Table S3) was positively correlated with the effector Treg signature in NSCLC patients from the TCGA (Fig. 4F). Thereafter, using the LegendPlex technology, we performed a thorough investigation of the cytokines commonly produced by CD4^+^ Treg and T helpers in the cocultures of naive CD4^+^ T cells with ECs. Notably, in contact with TNFα/IL-1ß-activated EC monoculture (cytokines-NECs), naive CD4^+^ T cells seem to polarize in Th2 (IL2^−^, IL5^+^, IL13^+^) and Th17 (IFNγ^+^, IL22^+^) (Figs. 4G-J; Supplementary Figure S5C). Our coculture results indicate that A549-TECs could induce the secretion of IL-2 and IL-5, secreted by Th1 and Th2 respectively (Fig. 4G-H). On the other hand, H1975-TECs only fostered IL-22 secretion, with low IL-17 levels suggesting a Th22 polarization (Figs. 4I-J) [36, 37]. Notably, IL-4, IL-13 and IFNγ showed minimal variation (Supplementary Figure S5C), and IL-9, IL-10 and IL-17 A were undetectable. Combination of various factors are described to induce polarization towards specific CD4^+^ subtype. For instance, IL-6 and TNF-α (without TGFß) known to stimulate the differentiation in Th22 [38], are upregulated in H1975-TECs (Fig. 4C; Supplementary Figure S5D). It is worth noting that, only the A549-TECs transcriptomic signature (Supplementary Table S3) appeared to be significantly associated with reduced survival in NSCLC patients suggesting that A549-TEC-mediated Treg or Th1/Th2 polarization could exert pro-tumor functions (Fig. 4K; Supplementary Figure S5E). Therefore, our data collectively suggest a Th22-polarizing effect of H1975-TECs and a mixed Th1/Th2 signature of A549-TECs, which may be important for patient survival and therapeutic response.

### NSCLC-TECs mediate the polarization of pro-tumor M2-like macrophages

**Fig. 5:**
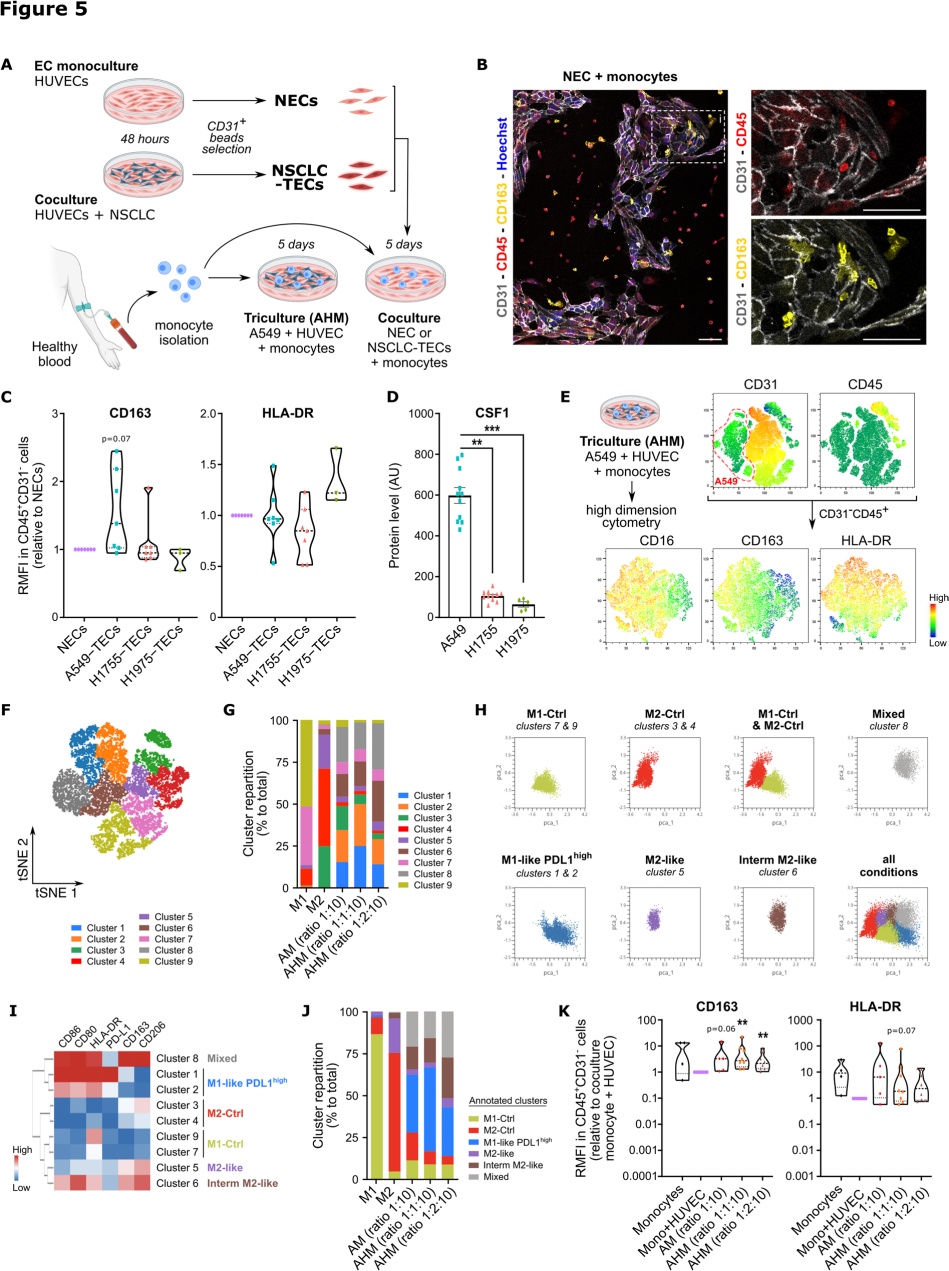
NSCLC-TECs induce the polarization of pro-tumor M2-like macrophages. **A**) Schematic representation of the 5 days coculture between PBMC-derived monocytes and NECs/NSCLC-TECs, and the AHM triculture between the A549 NSCLC line, HUVECs (NECs) and PBMC-derived monocytes. **B**) Confocal analysis of the coculture between NECs and monocytes, and stained for the endothelial marker CD31, the leukocyte marker CD45, and the M2-like macrophage marker CD163. Scale bar: 100μm. **C**) Flow cytometry analysis of M1-like (HLA-DR) and M2-like (CD163) polarization markers after the coculture between monocytes and NECs/NSCLC-TECs normalized to NECs. **D**) Mass spectrometry proteomic analysis of the secretome of NSCLC cell lines for CSF1. **E**) tSNE representations of the AHM triculture. The top right panels are gated on the CD31^-^CD45^+^ population to study macrophage polarization. **F**) tSNE representation of the different clusters identified (in the CD31^-^CD45^+^ population) in the AHM tricultures and in the M1-/M2-induced controls. **G**) Clusters repartition (% to total) in the different coculture conditions and M1 and M2 controls. **H**) PCA of the identified clusters in the M1, M2 controls and AHM triculture conditions.**I**) Heatmap of proteins levels in the different clusters identified. HLA-DR, CD80 and CD86 are M1-like macrophages markers while CD163, CD206 and PD-L1 are M2-like macrophages markers. **J**) Clusters repartition (% to total) of the grouped and annotated clusters in the M1, M2 controls and AHM triculture conditions. **K**) Relative mean of fluorescence (RMFI) of CD163 and HLA-DR in the CD31^-^CD45^+^ population after AHM triculture. Data are mean ± SEM, n >4, **p < 0.01, ***p< 0.001, Wilcoxon test compared to NECs or Mono/HUVEC

The NSCLC immune TME is mostly composed of myeloid cells including monocyte-derived macrophages, that have pivotal functions in tumor immunity [39]. To unravel the impact of ECs during this process, we cocultured NSCLC-TECs and NECs with monocytes isolated from healthy peripheral blood (Fig. 5A). After 5 days of culture, confocal microscopy analysis of monocyte/NEC cocultures revealed that CD45^+^CD163^+^ monocytes appear enriched in areas of close contact with NECs (Fig. 5B). We then further analyzed by flow cytometry monocyte/NEC or TEC cocultures, and particularly the CD31^−^CD45^+^ cell fraction for pro-tumor M2-like (CD163) and anti-tumor M1-like (HLA-DR) macrophage markers. Cocultures between NECs/TECs and monocytes had limited impact on monocyte polarization. Nonetheless, we observed a propensity toward an increased expression of CD163 and HLA-DR with A549- and H1975-TECs, respectively (Fig. 5C). Previous studies from our lab showed that thoracic cancer cell lines could mediate the M2-like polarization of monocytes in 2D and 3D cocultures, involving tumor-secreted cues including the M-CSF/CSF1 [40–42]. Interestingly, mass spectrometry analysis demonstrated a higher expression of CSF1 in the A549 secretome compared to those of H1755 and H1975 (Fig. 5D). Hence, to take the direct impact of NSCLC on monocyte polarization into account, we tricultured in 2D the A549 NSCLC cell line with ECs (HUVECs) and monocytes for 5 days (Fig. 5A). This triculture is referred to as the AHM triculture in the text. We then screened by flow cytometry the AHM triculture by gating on the CD31^−^CD45^+^ immune population that was well defined on t-SNE representations (Fig. 5E). Those cells segregated into 9 different clusters and distributed differently across conditions (Figs. 5F-G). Clusters 7 and 9 appeared specifically enriched (86% of all CD31^−^CD45^+^ cells) in M1-like control macrophages (M1-Ctrl), while clusters 3 and 4 were overrepresented (71%) in M2-like control macrophages (M2-Ctrl) (Fig. 5G). On the basis of PCA and correlation heatmap, clusters with similar marker expression profiles were grouped and annotated (Figs. 5H-J). The different 2D cocultures with the A549 NSCLC cell line led to high heterogeneity with the emergence of new phenotypes absent from the M1-/M2-controls: M1-like macrophages expressing high level of the immunosuppressive molecule PD-L1, a cluster with intermediate levels of M2-like markers, and a cluster with a mixed phenotype. Interestingly, increasing amounts of ECs in the AHM triculture expanded the mixed and intermediate M2-like clusters, when compared to the A549-monocyte coculture (Fig. 5J). When monocytes were co-cultured with A549, we reproduced the heightened CD163 expression as seen with other thoracic cancer cell lines [40, 42] (Fig. 5K). However, the addition of ECs in the AHM triculture led to significantly higher level of the M2-like marker CD163, while minimally affecting the M1-like marker HLA-DR (Fig. 5K). Altogether, our 2D coculture experiments indicate that ECs, when educated by NSCLC cells, could favor the emergence macrophage populations with pro-tumor M2-like traits.

### A multicellular tumor spheroid (MCTS) 3D model to mimic the lung TME

**Fig. 6:**
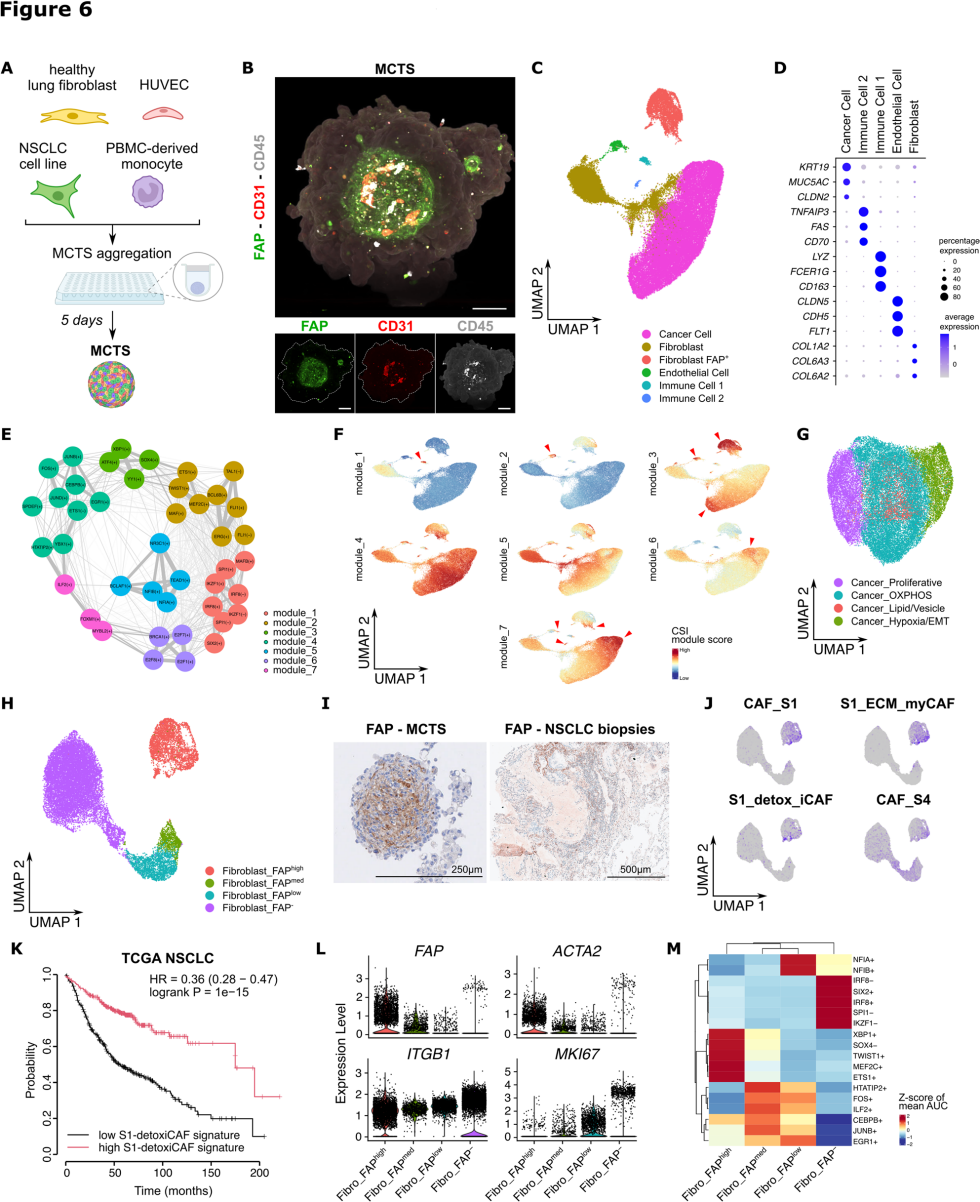
Characterization of the multicellular tumor spheroid (MCTS) model at the single cell level.**A**)Schematic representation of the MCTS formation during 5 days. **B**) Z-stack projection of confocal imaging in MCTS for the cancer-associated fibroblast (CAF) marker FAP, the leukocyte marker CD45 and the endothelial marker CD31. Scale bar, 100μm. **C**) uMAP representation of each cell type composing the MCTS and detected after scRNA-sequencing (n=4). **D**) Dotplot showing key marker genes for each cell type in the MCTS. **E**) Identification of 42 regulons organized in 7 co-expressions modules representing transcription factors and their targets. (+): activator, (-): repressor. **F**) UMAP representations of the UCell score of CSI modules depicted in panel **E**) with specific cell clusters identified (red arrow head). **G**) UMAP of the different sub-clusters identified in cancer cells. OXPHOS: oxidative phosphorylation; EMT: epithelial-to-mesenchymal transition. **H**) UMAP of the different sub-clusters identified in fibroblasts based on *FAP* expression. **I**) Immunostaining of FAP on MCTS (scale bar = 250µm) and NSCLC biopsy (scale bar = 500µm). **J**) UMAP of the different sub-clusters identified in CAFs thanks to gene signature from breast cancer scRNA-seq dataset. The sub-clusters are named CAF_S1, S1_ECM_myCAF, S1_detox_iCAF and CAF_S4. **K**) Survival probability of NSCLC patient using TCGA dataset depending on the high or low S1-detoxiCAF signature. **L**) Violin plot of expression level of key genes in the different fibroblasts sub-clusters. **M**) Heatmap of the expression of dysregulated transcription factors in the different fibroblasts sub-clusters. The Z-score of mean AUC represents the level of upregulation or downregulation. Heatmap only shows the top 5 most specific regulon per cell type. (+): activator, (-): repressor

Over the past decade, new 3D culture systems have emerged that account for the formation of a tumor model in its microenvironment. These systems are relevant and appear closer to an in vivo setting than classical 2D monocultures [43, 44]. Hence, in order to complexify our coculture systems, we modeled the TME with 3D MCTS. MCTS were obtained by spontaneous cell aggregation over 5 days with a defined ratio (2:1:1:1) of A549, HUVECs (NECs), PBMC-derived monocytes isolated from the peripheral blood of healthy donors, and primary healthy lung fibroblasts isolated from NSCLC patients (>5 cm away from the tumor) (Fig. 6A). As others have previously published [45], we demonstrated that fibroblasts are necessary for MCTS condensation and formation, likely through the secretion of essential extracellular matrix (ECM) components (data not shown). Confocal microscopy imaging revealed an elaborated cellular organization with FAP^+^ cells (a marker for cancer-associated fibroblast; CAF) forming a network in the center of MCTS, embedding clusters of CD31^+^ ECs and CD45^+^ monocytes where those cells appeared to form privileged contacts (Fig. 6B). To further unravel this complexity, MCTS were analyzed by single cell RNA-sequencing (scRNA-seq). After quality filtering, doublet removal and integration (Supplementary Figure S6A-D), we obtained 52,206 high-quality cells that clustered into 6 distinct clusters, annotated on the basis of their top-50 marker genes (Figs. 6C-D; Supplementary Figure S6E; Supplementary Table S4). Using transcription single-cell regulatory network inference and clustering (SCENIC), we could detect enriched activity of transcription factors for each cell cluster and sub-clusters. We identified 42 regulons that are organized into 7 major co-expression modules, that consists of transcription factors together with its predicted targets (Fig. 6E; Supplementary Figure S6F). When mapping the average activity score of each module onto uMAP, we found that each module refers to specific cell cluster/subclusters. For instance, module 1 associated with IRF2, SPI1 and IKZF1 transcription factors, important for macrophage polarization [46–49]. uMAP plot of module 2 signature indicates the endothelial cluster with pivotal transcription factors for vascular biology including ERG, FLI1 and TAL1 [50–53]. Finally, module 6 associated with E2F transcription factor members (E2F1/7/8) and BRCA1, which correlated with proliferative cells (Figs. 6E-F; Supplementary Figures S6G-I).

In this study, we focus primarily on stromal and immune cells from the TME rather than on cancer cells themselves, as using a single cancer cell line could hardly recapitulate patient tumor heterogeneity. Nevertheless, cancer cells were identified by the expression of epithelial cell markers (Supplementary Figure S6E) and genetic alterations as outlined by inferring somatic large-scale chromosomal copy number alterations with inferCNV (Supplementary Figure S7A). This step was necessary to formally distinguish cancer cells and fibroblasts that share similar features. The 31,878 cancer cells fall into 4 subclusters (Supplementary Figures S7B-E) with GSEA and marker genes showing specific transcriptomic signatures such as a proliferative subcluster, oxidative phosphorylation, lipid metabolism and vesicle secretion, and hypoxia/epithelial-to-mesenchymal transition (EMT) signaling (Fig. 6G; Supplementary Figures S7F; Supplementary Table S4). We then performed trajectory analysis starting with proliferative tumor cells, which reveals multiple differentiation pathways culminating in the hypoxic/EMT cluster (Supplementary Figure S7G). In summary, we demonstrate that the 3D MCTS model enables the emergence of multiple tumor cell phenotypes despite the use of a single NSCLC cell line.

### Normal fibroblasts appear educated by the TME in MCTS

Although primary healthy fibroblasts were initially used to form the MCTS, among the 19.389 fibroblasts identified, 33% expressed the CAF marker FAP at high (18%) or medium/low (15%) levels and this was observed in all samples (Fig. 6H; Supplementary Figures S8A-B). Conversely FAP^−^ fibroblasts displayed a higher variability between donors (Supplementary Figure S8A-B). Fibroblast sub-clustering was confirmed by plotting highly variable genes that correlated with FAP expression profile on UMAP (Supplementary Figures S8C-D). We further analyzed FAP expression using immunostaining on MCTS and NSCLC biopsies. There was localized expression at the center of the MCTS, and heterogeneous expression in NSCLC biopsies (Figs. 6B and I). Within MCTS, several fibroblast phenotypes were identified thanks to gene signatures from breast and lung cancer scRNA-seq data [54, 55], including immunosuppressive S1_myCAF (myofibroblast CAF), and a small cluster of immunoactivatory S1-detox-iCAF (Fig. 6J). A higher subcluster resolution was achieved after subsetting and reclustering FAP^high^ fibroblasts, which allow to identify new fibroblast populations including a cluster with: (i) enriched pathways related the unfolded protein response (UPR) and DNA-repair, (ii) a signature related to TNFα signaling, (iii) IFNγ-iCAF, and (iv) an uncharacterized cluster expressing several lncRNAs (Supplementary Figure S8E-I; Supplementary Table S4). Interestingly, only the S1-detox-iCAF signature correlated with improved survival in NSCLC TCGA (HR = 0.36), while the S1-ECMmyCAF signature did not associate with lower survival (Fig. 6 K; Supplementary Figure S8J). Moreover, based on the expression of *ACTA2* (αSMA) and *ITGB1* (CD29), we can tentatively assign the FAP^−^ cluster as *ITGB1*^high^*ACTA2*^high^ CAF S4 with perivascular/contractile properties and associated with invasion and metastasis [56] (Fig. 6L). Among the top 5 regulons, the FAP^−^ cluster expressed a high score for SIX2 [57], an important transcription factor for myofibroblast differentiation, and PU.1/Spi1 a pivotal regulator of fibroblast activation [58] that also correlated with proliferative capacity as shown by the increase *MKI67* expression in the FAP^−^ cluster (Figs. 6L-M). The FAP^high^ cluster was characterized by TWIST1, a key regulator of CAFs [59], and SOX4 and MEF2C two transcription factors aberrantly activated in myCAFs and promoting EMT [59, 60]. Altogether, our data highlight a high level of heterogeneity among fibroblasts within MCTS. This is likely due to their transcriptomic rewiring via cell-cell interactions with cancer cells but also immune cells and ECs in the TME.

### MCTS ECs adopt different phenotypes with homologies to NSCLC tumors

**Fig. 7:**
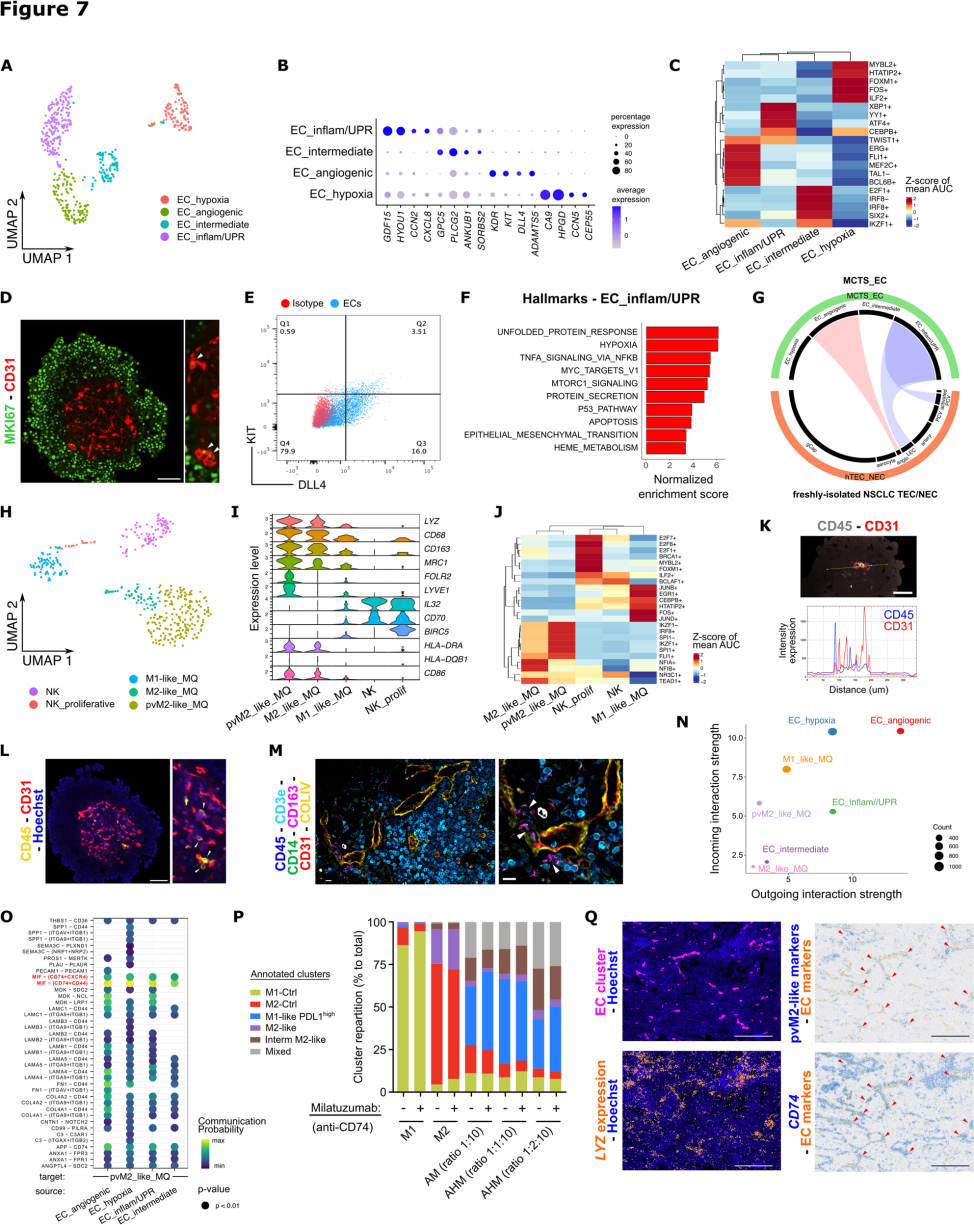
The MCTS as a 3D model to study EC-immune cell interactions in the tumor microenvironment.**A**) UMAP of the different sub-clusters identified in endothelial cells (ECs). UPR: unfolded protein response. **B**) Dotplot showing the expression of key marker genes for each EC sub-clusters. **C**) Heatmap of the expression of dysregulated transcription factors in the different EC sub-clusters. The Z-score of mean AUC represents the level of upregulation or downregulation. Heatmap only shows the top 5 most specific regulon per cell type. (+): activator, (-): repressor. **D**) Z-stack projection of confocal imaging in MCTS for CD31 and the proliferative marker MKI67. Scale bar, 100μm.** E**) Cytometry analysis of CD31^+^ cells from the MCTS for angiogenic markers KIT and DLL4, as compared to control isotypes. **F**) Gene set enrichment analysis of the upregulated pathways in the EC sub-cluster inflammatory/UPR. **G**) Circleplots showing similarities between MCTS-EC and freshly isolated TEC/NEC clusters. **H**) UMAP of the different immune cell sub-clusters. NK: natural killer; MQ: macrophage; pvMQ: perivascular macrophage. **I**) Violin plot of the key marker genes expressed in the immune sub-clusters. **J**) Heatmap of the expression of dysregulated transcription factors in the different immune sub-clusters. The Z-score of mean AUC represents the level of upregulation or downregulation. Heatmap only shows the top 5 most specific regulon per cell type. (+): activator, (-): repressor. **K**) Graph representing the intensity of expression regarding the distance between CD45^+^ and CD31^+^ cells. **L**) Confocal imaging of MCTS for CD31 and the immune cell marker CD45. Arrow heads indicate CD45^+^ cells located near CD31^+^ cells. **M**) Multiplex spatial proteomic on NSCLC human biopsies. Scale bar, 80μm. Asterix indicates an artefact that has been detected by comparing first and last blank cycles. Arrow heads indicate CD163^+^ M2-like macrophages. **N**) Cell-chat ligand-receptor predicting incoming and outgoing interactions between endothelial and macrophages sub-clusters. **O**) Communication probability of identified pathways between EC sub-clusters and the pvM2-like macrophage. Highly-statistical interactions related to the MIF pathway are indicated in red. **P**) Clusters repartition (% to total) in the CD31^-^CD45^+^ population from the A549-EC-monocyte (AHM) tricultures and in the M1-/M2-induced controls, treated or not with the anti-CD74 milatuzumab antibody. **Q**) Spatial transcriptomic on NSCLC human biopsy showing transcriptional expression of the macrophage marker *LYZ*, MIF-receptor *CD74 *and pvM2-like markers (*LYZ*,*FOLR2*, *CD163*). Arrow heads indicate co-expression of pvM2-like markers and *CD74* around vessels. Human Lung Cancer Visium HD dataset analyzed using Space Ranger 3.0.0. Scale bar, 500µm

With respect to ECs isolated from MCTS (coined MCTS-ECs), the 505 cells obtained post filtering segregated into 4 distinct subclusters with specific transcriptomic signatures (Figs. 7A-B; Supplementary Figures S9A-C; Supplementary Table S4). Presumably linked to the hypoxic nature of the central core of MCTS, we found a first hypoxic EC cluster characterized by the hypoxic marker *CA9* and the prostaglandin-degrading *HPGD* that was closely associated with capillary aerocytes in human lungs [61]. Probably linked to the hypoxic response, this MCTS-EC cluster also expressed established proliferation markers (*TOP2A*,* MKI67*,* BIRC5*, *CEP55*) and pathways related to ECM remodeling (Fig. 7B; Supplementary Figures S9D-E). A second MCTS-EC subcluster, coined angiogenic MCTS-ECs expressed genes involved in angiogenesis (*KDR*, *FLT1*, *TIE1*,* KIT*), established transcription factors (ERG, FLI1) [50, 52] and new ones (BCL6B) [62], tip-like markers (*INSR*, *ANGPT2*, *ANGPTL2*) and ECM remodeling (*COL4A1*,* COL4A2*,* COL18A1*), as described previously [9] (Figs. 7A-C; Supplementary Figure S9F). Of note, the proliferative MCTS-EC subcluster also expresses some of angiogenic markers, suggesting a stalk-like phenotype. A third MCTS-EC subcluster had a mixed intermediary phenotype with a poorly defined signature. It was thus coined as intermediate ECs (Figs. 7A-B; Supplementary Figure S9G). Using confocal imaging and flow cytometry we have validated some of the main markers from the proliferative and angiogenic MCTS-EC clusters (Figs. 7D-E). Finally, the fourth MCTS-EC subcluster displayed a marked pro-inflammatory signature with genes encoding for cytokines (*CXCL1*, *CXCL2*, *CXCL8*), NFKB pathway (*CCN2*), leukocyte adhesion molecule (*ICAM1*), endothelial-to-mesenchymal transition (*SERPINE1*), and endoplasmic reticulum stress-response (*HSP90B1*/GP96, *HSPA5*/BIP, *PDIA4*, *SELENOK/S*/*M*, *DNAJC*, *SERP1*, *HERP*,* HYOU1*) (Fig. 7A-B). The transcription factors ATF4 and XBP1, that are key signal transducers in the UPR and the endoplasmic stress responses, respectively, were also enriched in the inflammatory/UPR EC cluster (Fig. 7 C). GSEA further highlighted the implication of the UPR, autophagy and mTORC1 signaling in the inflammatory/UPR MCTS-ECs, as recently characterized in mouse TECs [11, 12] (Fig. 7F). Monocle trajectory analysis suggests a branching point between angiogenic and inflammatory/UPR ECs, when the proliferative/hypoxic cluster was selected as a starting point (Supplementary Figure S9H-J). We then sought to unravel the relevance of those MCTS-EC phenotypes by determining their similarities across models. While the proliferative/hypoxic and angiogenic MCTS-ECs were found in scRNA-seq datasets of 2D monoculture of NECs (HUVECs) [63], the inflammatory/UPR MCTS-EC subcluster was absent in early 2D culture of HUVECs (Supplementary Figures S9K-L). Conversely, the inflammatory/UPR MCTS-EC subcluster was expressed in freshly-isolated ECs from NSCLC human biopsies and appeared similar to ECs with immunomodulatory functions such as lymphatic ECs and activated postcapillary venule (Fig. 7G; Supplementary Figure S9M). Likewise, the inflammatory/UPR MCTS-EC subcluster was analogous to a cluster expressing a mesenchymal/endothelial-to-mesenchymal (EndMT) signature found in 2D culture of TECs from NSCLC biopsies [9] (Supplementary Figures S9N-O). In contrast to culture conditions (and LLC mouse NSCLC tumors), no proliferative EC cluster was identified in human NSCLC biopsies. Finally, we compared endothelial transcriptomic signatures from our MCTS model and the NSCLC-TEC 2D coculture data. Interestingly, the greatest number of similarities was found between NSCLC-TECs and the hypoxic EC cluster. These similarities involved genes associated with the prostaglandin pathway (*PTGES*, *AKR1B10*, *AKR1C2*, *AKR1C1*), inflammation (*IL18*), complement system (*C1R*, *C3*, *F12*), ECM and matrix remodeling (*MMP7*, *S100P*, *SPP1*, *HPGD*, *S100A4*), coagulation and thrombosis (*FGA/B/G*, *FGL1*, *PLAU*, *SERPINA1*) and cell adhesion (*DSP*, *MUC5B*, *FMN1*, *LAMB3*) (Supplementary Table S5). Therefore, the endothelial compartment within MCTS exhibits a high level of heterogeneity, including specific EC clusters that resemble EC populations found in NSCLC. As such, these clusters may have therapeutic relevance.

### MCTS enables the rewiring of monocyte-derived macrophages and their interactions with ECs

We then further phenotyped the immune cells of MCTS clusters, originating from PBMC-derived circulating monocytes. Subclustering analysis revealed an interesting heterogeneity with 434 cells that cluster into 5 subclusters (Fig. 7H). Notably, probably due to contamination inherent to the monocyte isolation, 2 small clusters of natural killer (NK) cells were identified, expressing *CD96*, *IL32*,* CD70* and *RELB* (Figs. 7H-I). They were labeled as NK, and NK proliferative by interrogating reference-based atlas [64]. Those (under-represented) lymphoid clusters will not be further analyzed in this study. Emphasizing on macrophage diversity owing to differentiation and activation processes in the TME, 3 heterogeneous clusters of myeloid cells were identified on the basis of canonical markers (*LYZ*, *CD68*) (Fig. 7I). Although a continuum exists within these subsets, we detected (i) a perivascular M2-like macrophage (pvM2-like MQ) characterized by *CD163* and the accepted markers *LYVE1*, *FOLR2*, *SIGLEC1* (CD169) and *VSIR* (VISTA) [65]; (ii) a M2-like phenotype with high expression of M2-like markers but no expression of *LYVE1* or *FOLR2*; and (iii) a population of M1-like macrophage expressing low levels of the aforementioned markers (Fig. 7I). Notably, MCTS lacking the endothelial compartment showed significantly reduced levels of the pvM2-like MQ markers *LYVE1*, *FOLR2* and *CD163* (Supplementary Figure S10A). Consistent with our 2D coculture models (Fig. 5F-I), monocytes placed in a tumor-like microenvironment appear to adopt phenotypes that differ from classical M1 and M2 polarization. Nevertheless, several transcription factors regulating inflammatory genes are associated to the M1-like macrophage cluster including EGR1 [66] and the AP-1 transcription factors junD (JUND), junB (JUNB) and c-fos (FOS) [67] (Fig. 7J). On the other hand, the transcription factor TEAD1 is predicted to be activated in the M2-like macrophage cluster, and recently described to contribute to alternative TGFß-mediated macrophage polarization in kidney fibrosis [68] (Fig. 7J). Interestingly, the M2-like fraction represents 77% of the myeloid compartment, mirroring our previous observations in NSCLC and mesothelioma MCTS that most monocyte-derived macrophages adopt an M2-like phenotype [41, 42]. Given that CD45^+^ cells were found in close proximity to ECs in MCTS (Figs. 6B, 7K-L), and that we also identified CD45^+^CD163^+^ cells close to CD31^+^ vessels in NSCLC tumor biopsies by multiplex spatial proteomic detection (Fig. 7M), we used CellChat to predict ligand-receptor interactions between MCTS macrophages and EC subsets, in order to identify therapeutically relevant targets. Notably, the angiogenic MCTS-EC subcluster was predicted to perform most interactions (both incoming and outgoing), with the highest weight (Figs. 7N; Supplementary Figure S10B). We then analyzed global communication patterns (incoming or outgoing) across cell clusters considered. Intriguingly, while the angiogenic, inflammatory/UPR and hypoxic ECs were predicted to have very similar communication patterns when considered receiver cells, they show sharp differences as sender cells (Figs. 7N; Supplementary Figures S10C-D). Among those pathways, the angiogenic ECs seems to contribute with molecules related to the Angiopoietin (ANGPT) and Ephrin-ß (EPHB) family known to be important during cell migration and chemotaxis (Supplementary Figure S10C). Interestingly, the macrophage migration inhibitory factor (MIF) pathway showed the most significant communication probabilities between ECs and macrophage clusters, and particularly for the pvM2-like macrophages as an incoming signal (Fig. 7O; Supplementary Figure S10E). MIF was identified as a ligand of both CXCR2 and CXCR4, that are major regulators of inflammatory cell recruitment [69]. Moreover, another MIF receptor is CD74, involved in TNF-α–dependent lung inflammation [70], and highly expressed at the transcriptional level in M2-like macrophage subsets (Supplementary Figure S10F). To functionally validate the relevance of this pathway we treated AHM triculture with milatuzumab, a humanized monoclonal anti-CD74 antibody, and studied monocyte polarization. Consistent with previous report in various cancer types, MIF targeting reduced M2-like polarization and increased the proportion of M1-Ctrl and M1-like PDL1^high^ macrophages (Fig. 7P; Supplementary Figures S10G-H). By reanalyzing spatial gene expression dataset from NSCLC biopsies, we could further validate that cell foci expressing the myeloid marker LYZ were localized around blood vessels. Moreover, a fraction of those myeloid cells co-expressed pvM2-like markers (*LYZ*, *CD163*, *FOLR2*) and the MIF receptor CD74 (Fig. 7Q). Altogether, this data supports the evidence that MCTS-ECs could regulate tumor macrophage polarization via various pathways, either directly or via a third partner, and that MCTS appears as a relevant model to mimic some aspects of cell-cell communications from the NSCLC TME.

## Discussion

The aim of our study was to model the TME and analyze the impact of ECs on various immune populations, in order to better dissect their immunoregulation function in the context of lung cancer. We showed that coculturing ECs with various NSCLC cell lines induced profound alterations of ECs at the transcriptomic, proteomic and kinomic levels with the induction of pro-inflammatory pathways, reflecting their activation state. Although we previously demonstrated that NSCLC-secretome could impact EC biology by inducing EndMT in HUVECs [24], the pro-inflammatory trait may require direct cell-cell contact as indirect coculture with inserts had minimal effect. Besides, this feature does not appear restricted to the NSCLC entity, as our meta-analysis revealed a similar transcriptomic signature in breast cancer cell cocultured-ECs. Despite a limited impact of NSCLC-TECs on CD8^+^ T lymphocyte proliferation and activation, we showed that CD4^+^ T cells were polarized into different subsets (Treg, Th22 and Th1/Th2) when cocultured with NSCLC-TECs. For the first time, we brought to light that OX40L could play a role in the immunoregulatory function of TECs, where it appears downregulated as compared to NECs. We also discovered that NSCLC-TECs could enhance the expression of M2-like markers.

In the search to improve our 2D coculture system, we scrutinized at the single cell resolution, 3D MCTS encompassing tumor cells, ECs, healthy fibroblasts and monocyte-derived macrophages. Overall, we showed that cell heterogeneity was much more complex than 2D cultures, with several cell phenotypes identified. For example, although healthy primary fibroblasts were used, we identified CAF subtypes, including pro-tumor S1-ECM myCAFs and anti-tumor S1-detox iCAFs. The former were much more prevalent than the latter, but suggesting a possible emergence of CAFs in 3D MCTS. Moreover, 3 subclusters of MCTS-ECs were distinguished, among which the inflammatory subcluster that was absent from standard 2D culture but present in freshly-isolated ECs from NSCLC biopsies. Finally, we uncovered various macrophage polarization states within NSCLC-MCTS, with a perivascular M2-like subset that is therapeutically important due to its interaction with the vascular compartment.

Our results on OX40L raise several outstanding questions and promising future perspectives. OX40L/gp34 belongs to the TNF family and is identified as the ligand for the costimulatory receptor OX40. Importantly, the use of an anti-OX40 agonist antibody has been shown to enhance T lymphocyte-mediated antitumor immunity and inhibit Treg induction in several cancer models, thereby promoting tumor regression and ameliorating patient survival [71, 72]. Moreover, OX40L has been identified to be expressed in ECs (HUVECs) where it can act as a co-stimulation signal for T cell recruitment and proliferation [30, 73, 74]. Here we show that OX40L expression was blunted in A549- or H1975-TECs, and their coculture with polyclonal CD4^+^ T cell inhibits lymphocyte proliferation following activation, while increasing Treg proportion. We hypothesize that inhibition of CD4^+^ T cell proliferation results from the failure of the proliferative support signal normally provided by OX40/OX40L at the time of T cell activation. Indeed, this hypothesis is reinforced by the fact that no difference in proliferation was observed in naive T cells, which only express OX40 very late after activation [75]. An important interrogation holds in deciphering the signaling leading to reduced OX40L transcriptomic and protein expression in some NSCLC-TECs compared to NECs. In various immune cell types, the OX40/OX40L signaling relies on the NFκB pathway [33–35]. Moreover, in HUVECs the OX40 binding results in c-Jun and c-Fos induction [76] that closely interact with the NFκB p65 subunit, thereby mutually activating their binding to DNA motifs [77]. Here, we demonstrate that the kinase activity of Lyn and Fyn, which are involved in the regulation of the NF-κB pathway [78], is reduced in A549- and H1975-TECs. This reduction could be implicated in the diminution of OX40L, but this hypothesis requires further testing. Noteworthy, given the role of the OX40/OX40L interaction in the establishment of an effector memory response [31], it will be relevant to examine effector (CD45RA-CCR7-) and central (CD45RA-CCR7^+^) memory T cell markers after activation upon coculture with NSCLC-TECs. Finally, recent evidence indicates that endothelial OX40 counteracts the antitumor effects produced in T cells by promoting angiogenesis in colorectal cancer [79]. In a previous study we have shown that OX40 is a marker of the angiogenic EC subtype in NSCLC biopsies [9]. However, our current set of data in vitro and TCGA datasets suggest a different role in NSCLC, with a potential context-dependent role for OX40L/OX40 pathway in EC that warrant further investigations.

The characterization of IL-22 Th22 cells is relatively recent and initially defined as cells specializing in tissue repair processes. Intra-tumor IL22-producing Th22 cells, which proportion is increased in tumor tissues, is associated with poor prognosis in several cancer types [80–82]. Here we show that naive CD4 T cells cocultured with H1975-TECs fostered IL-22 secretion, while producing low IL-17 levels and thus suggesting a Th22 polarization [36, 37]. However, the effect of A549-TECs appears less clear compared to the control condition stimulated with TNFα/IL-1ß where naive CD4^+^ T cells polarize in Th2. Indeed, although IL-5 secretion was induced upon coculture with A549-TECs, there was only a tendency toward heightened IL-13, and the IL-2 (a Th1 marker) was also induced. We hypothesize that this incomplete Th2 signature might relate to the coculture duration and would require later end-point analysis. A mass spectrometry proteomic characterization of NSCLC-TECs secretome may also identify polarization mediators eventually involved in this mixed Th2 phenotype. Placing our results back in the context of the TME, we thus hypothesize that TECs may contribute to the polarization of Th2 and Th22 cells, hence participating in tumor immunosuppression. Following on from this project, it would be interesting to deepen our understanding of the underlying mechanisms involved in the interaction between ECs and Th2/Th22 cells in normal and tumor contexts.

In NSCLC, as it is the case for many other cancer entities, TAMs represent the major immune cell type, accounting for more than one third of tumor-infiltrating immune cells [39]. Macrophages display high intrinsic plasticity and adaptability based on epigenetic regulation and various cues emanating from the TME. Beyond the canonical M1/M2 macrophage phenotype dichotomy, scRNA-seq emphasized macrophage diversity in cancers [83]. As such, macrophages exist along a phenotypic continuum and are not easily characterized by individual markers [84, 85]. As shown by our lab and others, monocytes differentiate toward M2-like macrophages when cultured in 3D with tumor cells [40–42]. Here, we show by scRNA-seq a much deeper resolution of this macrophage continuum with several intermediate phenotypes. In 2D, when cultured with ECs, macrophages seem to express high levels of CD163 when located nearby ECs. Seemingly, in MCTS CD45^+^ monocytes appeared to form privileged contact with MCTS-ECs. These observations are consistent with the results from Njock et al. which indicate that treatment with extracellular vesicles produced by HUVECs during coculture with MDA-MB-231 induced polarization towards an M2 phenotype [19]. Finally, our MCTS data identify the presence of LYVE1^+^ FOLR2^+^ perivascular M2-like macrophages described to have therapeutic relevance by influencing treatment resistance, angiogenesis, EC-leukocyte interactions and patient survival [86–89]. Although protein validations will be required to firmly establish their true nature, it will be interesting to determine whether the presence of ECs alone suffices to induce M2-like perivascular traits or whether it requires other signals from the TME.

We acknowledge limitations of our study. First, we restrained our 2D and 3D coculture systems to two EC types with the HUVEC and HMVEC-L, but ECs from other vascular beds (arteries) may show different responses to the contact with tumor cells. For instance, we and others [9] showed that normal aerocyte and general capillaries appear downregulated in NSCLC tumors. As such tumor cells or the TME may have a particular detrimental effect on this EC type. Second, in our study we determined the effect of NECs/TECs on the tumor immune compartment, as well as how tumor cells could influence ECs during this process. In fact, cellular communication within the TME is not unidirectional, and lymphocytes, macrophages or fibroblasts also have impacts on ECs [90, 91]. Moreover, it will be important to explore cell heterogeneity when MCTS are devoid from the endothelial compartment. In order to make a reliable assessment of which cell phenotypes are potentially due to the presence of ECs. Third, cell-cell prediction including the MIF pathway will require to be thoroughly validated to dissect its precise immunoregulatory function within the TME. Fourth, the 2D cocultures and MCTS cannot be seen as ideal models to mimic the TME. Rather they are means to study specific cell type/phenotype in which this culture model appears similar to the in situ condition. Nevertheless, adding flow to the MCTS with microfluidic devices could certainly increase EC heterogeneity with artery/vein specification. Fifth, high-resolution spatial multiomics on MCTS could improve our understanding of cell-cell interactions and priming in the TME, cellular niches (e.g. hypoxic, vascular, etc.) and the impact of immunotherapies thereof. Notwithstanding these limitations, we believe our various culture models allow to better delineate the function of TECs within the TME, and how they could modulate tumor immunity. In the future, MCTS could be a valuable platform for drug screening and in-depth analysis of immune-related EC functions.

## Conclusion

The current study describes novel in vitro approaches to unravel how ECs are altered by cancer cells, and how these alterations could impact on tumor immunity. Our findings indicate that NSCLC-TECs influence immune responses by promoting immunosuppressive T cell (Th22, Treg) and M2-like macrophage polarization. Through the use of 3D MCTS, spatial and single-cell transcriptomic analyses, our study underscores the intricacy of EC-immune interactions and identifies TECs as pivotal regulators of tumor immunity. In particular, OX40L and MIF signaling emerge as interesting factors in TEC immunoregulation. Future exploration of these models is promising for evaluating new therapeutic strategies for NSCLC patients.

## Supplementary Information

Supplementary Material 1.

Supplementary Material 2.

Supplementary Material 3.

Supplementary Material 4.

Supplementary Material 5.

Supplementary Material 6.

## Data Availability

The dataset generated in this study is available via Zenodo at https://doi.org/10.5281/zenodo.17409889.

## Abbreviations

APC: Antigen Presenting Cell
CAF: Cancer-Associated Fibroblast
EC: Endothelial Cell
EMT: Epithelial-to-Mesenchymal Transition
EndMT: Endothelial-to-Mesenchymal Transition
GSEA: Gene Set Enrichment Analysis
HMVEC-L: Human Lung Microvascular Endothelial Cell
HUVEC: Human Umbilical Vein Endothelial Cell
MCTS: Multicellular Tumor Spheroids
MIF: Macrophage Migration Inhibitory Factor
NEC: Normal Endothelial Cell
NSCLC: Non-Small Cell Lung Cancer
PCA: Principal Component Analysis
PBMC: Peripheral Blood Mononuclear Cells
pvM2-like MQ: Perivascular M2-like Macrophage
SCENIC: Single-Cell Regulatory Network Inference and Clustering
scRNA-seq: Single Cell RNA-Sequencing
TEC: Tumor Endothelial Cell
TME: Tumor Microenvironment
UPR: Unfolded Protein Response
