# Supplementary material for: In vitro models to mimic tumor endothelial cell-mediated immune cell reprogramming in lung adenocarcinoma
Source: J Exp Clin Cancer Res. 2025 Nov 27;45:15. doi: 10.1186/s13046-025-03576-4 (PMC12817544; doi:10.1186/s13046-025-03576-4)
Supplement: Supplementary file 6 — Supplementary Material 6. [file 13046_2025_3576_MOESM6_ESM.docx]

**Supplementary material for**

**In vitro models to mimic tumor endothelial cell-mediated**

**immune cell reprogramming in lung adenocarcinoma**

Morgane Krejbich^1^, Emilie Navarro^1^, Judith Fresquet^1^, Marine Cotinat^1^, Valentin Isen^2^, Hortense Perdrieau^1^, Virginie Forest^3^, Aurélie Doméné^4^, Tiphaine Delaunay^1^, Hala Awada^1^, Vincent Dochez^5,6^, David Roulois^2^, Nicolas Boisgerault^1^, Richard Redon^3^, Christophe Blanquart^1^, Isabelle Corre^1^, Lucas Treps^1^

^1^Nantes Université, INSERM UMR 1307, CNRS UMR 6075, Université d'Angers,
CRCI2NA, F-44000 Nantes, France. ^2^UMR 1236, Univ Rennes, INSERM, Établissement Français du Sang, Équipe Labellisée Ligue contre le cancer, F-35000 Rennes, France. ^3^Nantes Université, CNRS, Inserm, l'institut du thorax, F-44000 Nantes, France. ^4^Nantes Université, CHU Nantes, CNRS, Inserm, BioCore, US16, SFR Bonamy, F-44000 Nantes, France. ^5^Nantes Université, CHU Nantes, Service de Gynécologie-Obstétrique, INSERM, CIC 1413, F-44000 Nantes, France. ^6^Nantes Université, CHU Nantes, Movement - Interactions - Performance, MIP, EA 4334, F-44000 Nantes, France.

**Corresponding author:** Lucas Treps, [lucas.treps@univ-nantes.fr](mailto:lucas.treps@univ-nantes.fr)

**Supplementary methods**

*SCENIC analysis*

The raw count matrix from Seurat object is exported to loom file with SCopeLoomR (v 0.13.0). Then, pySCENIC (v 0.12.1 with python v 3.11.5) grn and ctx step was run 10-time to address the stochastic nature of the gradient boosting regulon inference (according to the published nature protocols article ^1^). The required databases used for this step were found on <https://resources.aertslab.org/cistarget/> (“allTFs_hg38.txt” list of TFs for grn and cisTarget 2022 v10 SCENIC+ motif annotation with proximal TSS +500/-100 and distal TSS+/-10kb ranking database for ctx). The aggregation of ctx motifs from all runs was done accordingly to the archived Nextflow pipeline (10.5281/zenodo.3703108). We filtered the regulons with at least 80% of occurrences across all runs and 5 genes minimum according to the defaults of the pipeline. Regulons activity was inferred on cells using pySCENIC function aucell with default values (AUC threshold of 0.05). The AUC matrix was then added back to the Seurat object using CreateAssayObject function.

The CSI score was reimplemented from scratch similarly to the original publication ^2^ and used in the same way as in Suo et al. ^3^. Briefly, we first calculated the Pearson correlation coefficient (PCC) between each pair of regulons. Then, we defined a threshold for each pair of regulons equal to the PCC of the pair minus 0.05 and counted all regulons connected to either regulon of the pair with a PCC superior or equal to the threshold (excluding either regulon of the pair with itself). The CSI score for this pair is finally calculated as one minus the ratio of counted regulons on the total number of regulons. Optimal CSI modules determination is based on hierarchical clustering according to agreement of silhouette and elbow method on factoextra (v. 1.0.7). CSI modules are scored on each cell with UCell function AddModuleScore_UCell (v. 2.8.0)^4^ to aggregate AUC regulons score. The RSS score was adapted from the code of the R version of SCENIC (v. 1.1.2) based on the article of Suo et al. ^3^ and is defined as one minus the Jensen-Shannon distance of scores grouped by cell types. The top 5 RSS scores per cell types are used to select the most specific regulons per cell types.  Matrices were made using ComplexHeatmap (v 2.20.0)^5^.

*Secretome proteomic analysis*

Digestions and LC-MS/MS analyses were performed at the Prot’ICO proteomics facility. NSCLC cell lines were incubated 72 hours in RPMI with 10% SVFd and 1% P/S. Supernatants were washed away with 1X PBS and replaced by RPMI without SVF and allowed to condition this new medium for 24 hours. Cells were counted for subsequent normalization, and media (~6 mL) were collected and centrifuged 5 min at 300 x g and concentrated on an amicon spin reverse 5 kDa/2mL membrane filtration device. Proteins were denatured in 0.1% Rapigest SF™ acid-labile detergent (Waters^®^), 5 mM DTT and 50 mM ammonium bicarbonate, at 95°C for 30 min (200 µL final). Thiol residues were thus chemically reduced and subsequently cooled down to RT then protected by alkylation in 20 mM MMTS (Sigma-Aldrich) for 10 min at 37°C. 5µg of trypsin (ABSciex) per sample were added and incubated at 37°C overnight. Peptides were then cleared by centrifugation, desalted on C_18_ sep-pack reverse phase microcolumns as described in the “Stage-tips” procedure and peptides were eluted in Acetonitrile (ACN)^23^. Eluates were dried in a vacuum centrifuge concentrator (ThermoFischer Scientific), resuspended in 25 µL of 10% ACN and 0.1% Formic Acid (FA). Eluate flow was electrosprayed into a timsTOF Pro 2 mass spectrometer (also from Bruker®) for the 60 min duration of the hydrophobicity gradient ranging from 99% of solvent A (0.1% FA in milliQ-grade H_2_O) to 40% of solvent B (80% ACN and 0.1% FA in mQ-H_2_O). The mass spectrometer acquired data throughout the elution process and operated in data-independent analysis mode (DIA) with PASEF-enabled method using the TIMS-Control v.3.1.4 software (Bruker^®^). Samples were injected in batch replicate order to circumvent possible technical biases.

LC-MS/MS data analysis: The raw data were extracted, normalized and analyzed using Spectronaut software v. 18.6.231227.55695 (Biognosys) in Direct-DIA mode, which modelized elution behavior, mobility and MS/MS events based on the Uniprot/Swissprot sequence 2022 database of human proteins. Protein identification false discovery rate (FDR) was restricted to 1% maximum, with match between runs (MBR) option enabled and inter-injection data normalization.

**Supplementary references**

1. Van de Sande B, Flerin C, Davie K, et al. A scalable SCENIC workflow for single-cell gene regulatory network analysis. *Nat Protoc*. 2020;15(7):2247-2276. doi:10.1038/s41596-020-0336-2

2. Fuxman Bass JI, Diallo A, Nelson J, Soto JM, Myers CL, Walhout AJM. Using networks to measure similarity between genes: association index selection. *Nat Methods*. 2013;10(12):1169-1176. doi:10.1038/nmeth.2728

3. Suo S, Zhu Q, Saadatpour A, Fei L, Guo G, Yuan GC. Revealing the Critical Regulators of Cell Identity in the Mouse Cell Atlas. *Cell Rep*. 2018;25(6):1436-1445.e3. doi:10.1016/j.celrep.2018.10.045

4. Andreatta M, Carmona SJ. UCell: Robust and scalable single-cell gene signature scoring. *Comput Struct Biotechnol J*. 2021;19:3796-3798. doi:10.1016/j.csbj.2021.06.043

5. Gu Z, Eils R, Schlesner M. Complex heatmaps reveal patterns and correlations in multidimensional genomic data. *Bioinformatics*. 2016;32(18):2847-2849. doi:10.1093/bioinformatics/btw313

**Supplementary figures**


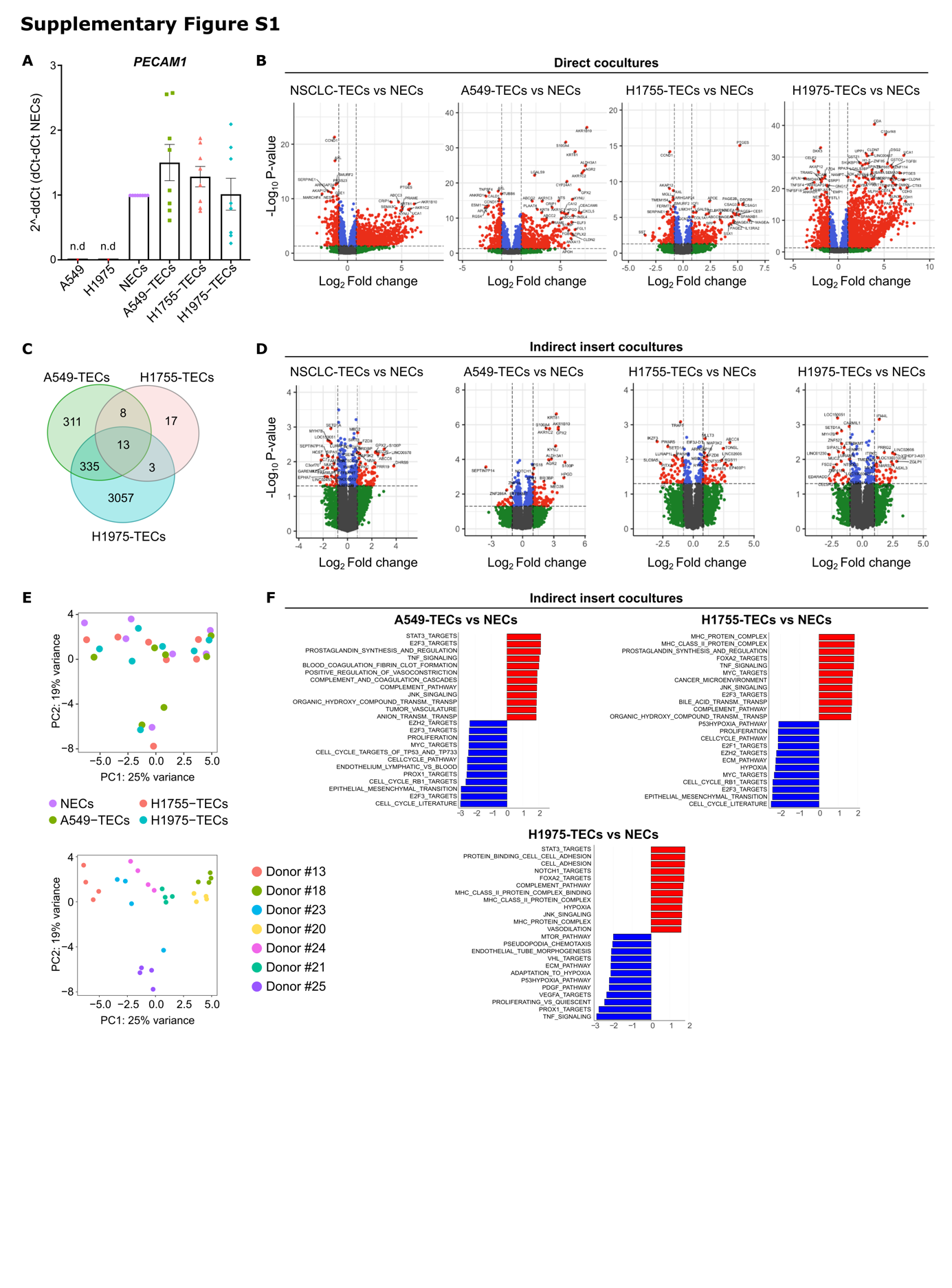


**Supplementary Figure S1 – Comparison of the direct and indirect 2D-coculture models.** A) RT-qPCR analysis for the endothelial marker *PECAM1* of NSCLC cell lines and NSCLC-TECs after enrichment. B) Volcano plots depicting differential analysis between each individual NSCLC direct coculture relative to the HUVEC monoculture (NECs). Adjusted p-value p<0.05. C) Venn diagrams indicating congruent deregulated genes between each NSCLC-coculture *versus* NECs (adjusted p-value p<0.05). D) Volcano plots depicting differential analysis for the indirect cocultures using inserts. E) PCA showing the distribution of each sample in the indirect coculture assay. Clustering appears mediated by the different HUVEC donors (lower panel) and not by experimental conditions (upper panel). F) Gene set enrichment analysis in the indirect insert coculture from each individual NSCLC-TECs *versus* NECs. Pathways enriched in each NSCLC-TECs appear in red (p-value p<0.05).


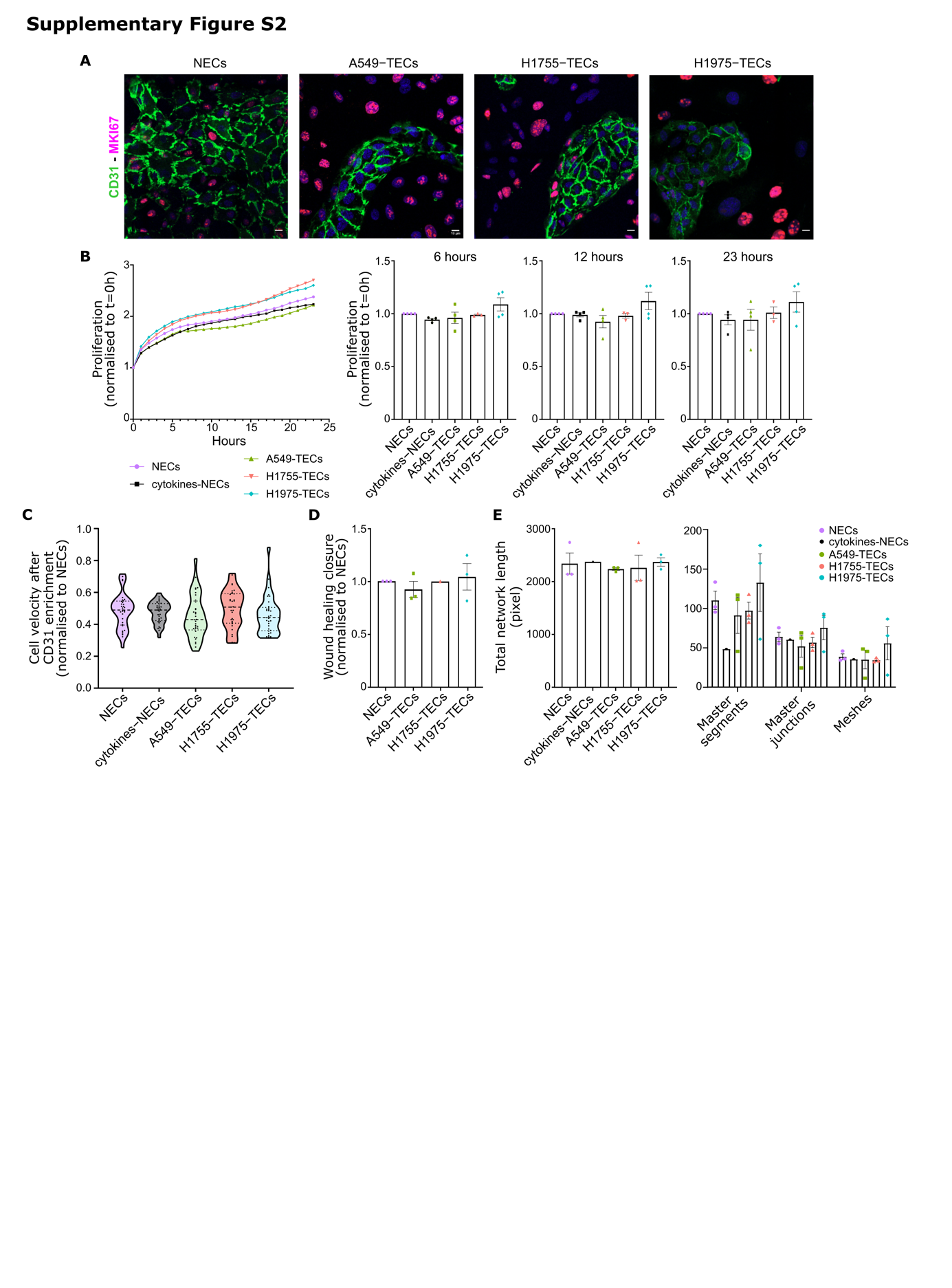


**Supplementary Figure S2** **– The impact of 2D-direct coculture on EC proliferation and migration.** A) Confocal microscopy of NECs and NSCLC-TECs cocultures, stained for the endothelial marker CD31 and the proliferation marker KI67. NECs and NSCLC-TECs were enriched with CD31 after cocultures and screened for various functions upon culture. Scale bar: 10µm. B) Proliferation, normalized to t = 0h in function of time and at different times (t = 6h, 12h and 23h) normalized to NECs. C) Velocity in μm/min and normalized to NECs. D) Scratch wound closure, and E) 2D tubulogenesis assay. Cytokines-NECs represent NECs treated with TNFα/IL-1ß. Data are mean ± SEM, n > 3, Wilcoxon test compared to NECs.


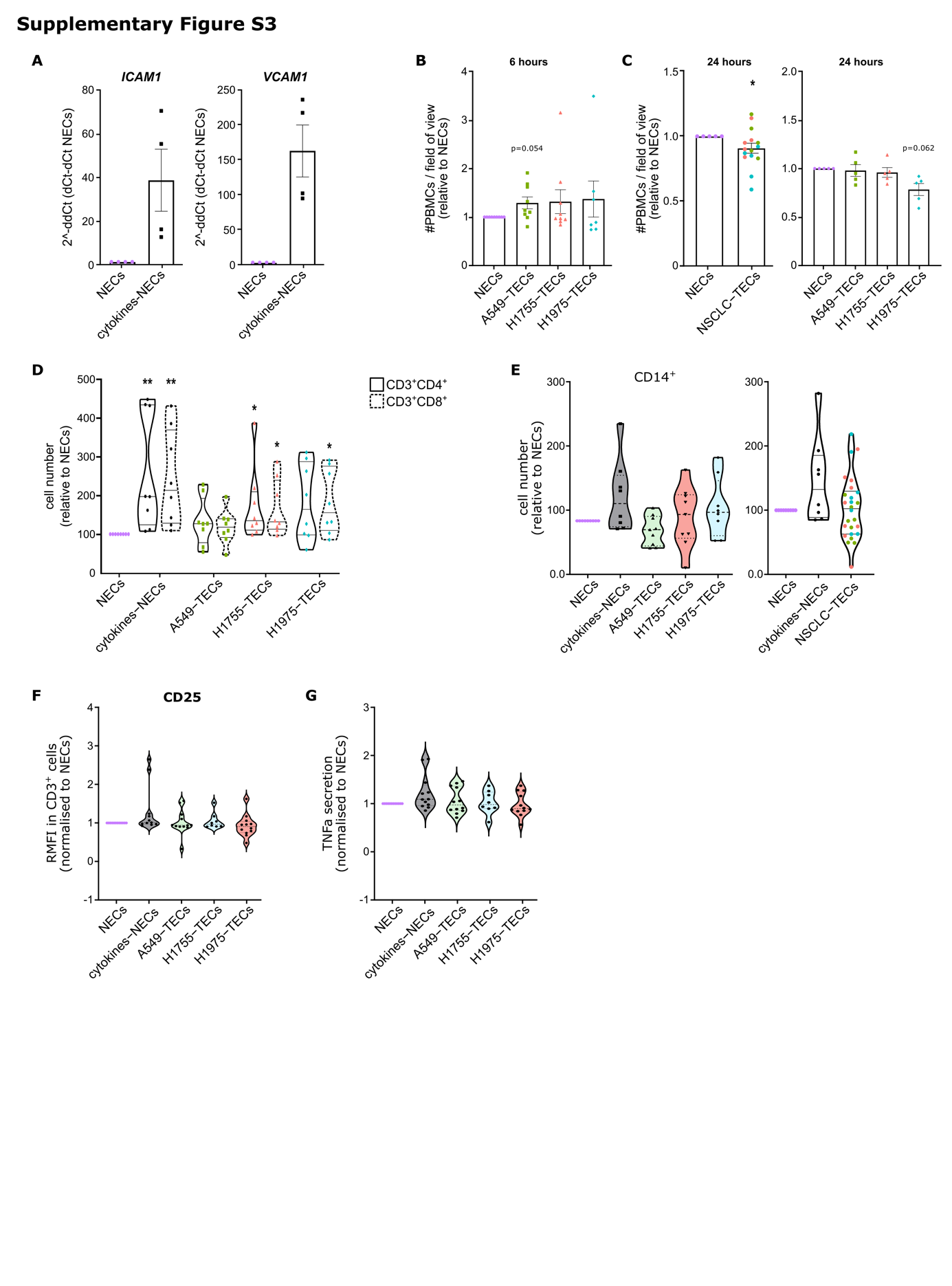


**Supplementary Figure S3** **– The impact of NSCLC-TECs on leukocytes and monocytes transmigration and polarization.** A) RT-qPCR analysis of NECs and NECs stimulated with TNFα/IL-1ß (cytokines-NECs) for *ICAM1* and *VCAM1*. B-C) NECs and NSCLC-TECs were cultured at confluency for B) 6h or C) 24h after coculture, and leukocyte adhesion assessed. D-E) Chemotaxis experiment for D) CD3^+^CD4^+^ cells and E) CD14^+^ monocytes attracted by the coculture medium from NECs alone or NSCLC-TECs. F) Flow cytometry analysis for CD25 and G) TNFα ELISA secretion in polyclonal activated CD8^+^ T cells cocultured with NECs or NSCLC-TECs. RMFI: Relative mean of fluorescence. Data are mean ± SEM, n > 8, *p < 0.05, **p < 0.01, Kruskal-Wallis test (A), and Wilcoxon test (B-G) compared to NECs.


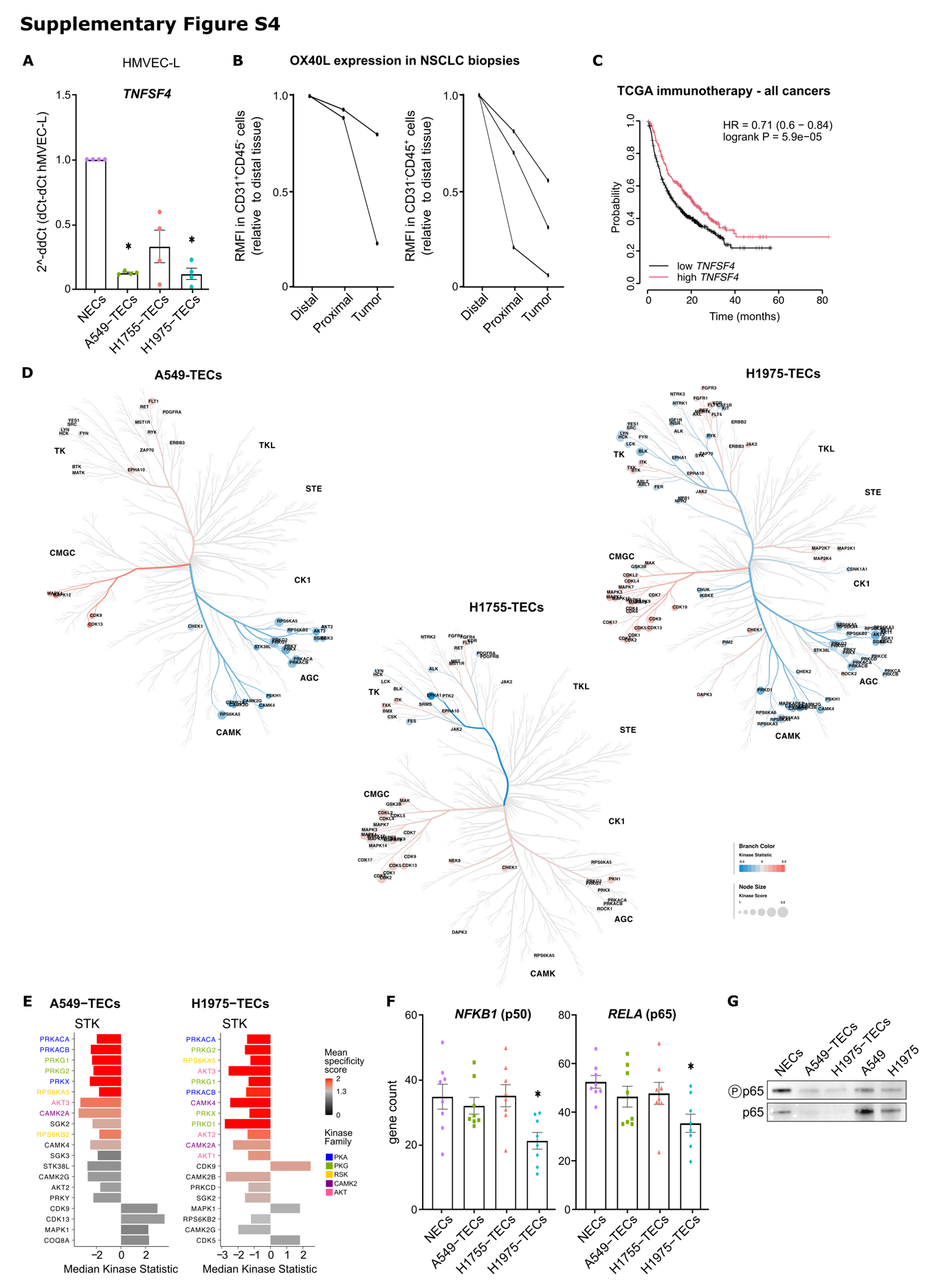


**Supplementary Figure S4** **– OX40L expression in lung cancers and kinome analysis of NSCLC-TECs.** A) RT-qPCR analysis of coculture experiments with HMVEC-L with or without NSCLC cells. B) Flow cytometry analysis for OX40L in CD31^-^CD45^+^ and in CD31^+^CD45^-^ isolated from NSCLC human biopsies harvested at different location from the tumor excision site, and expressed as relative to distal tissue (>5 cm is considered as non-tumoral). C) Survival probability of cancer patients treated with immunotherapies using TCGA dataset depending on the high or low TNFSF4 expression. D) Phylogenetic coral trees of the kinomic assay showing the kinases with deregulated activities for A549-TECs, H1755-TECs and H1975-TECs. E) Score plot analysis of protein serine threonine kinases differentially regulated in NECs compared to A549-TECs and H1975-TECs. A negative median kinase statistic means a downregulated kinase compared to NECs, and a specificity score in red is statistically significant. Kinase families are also represented in blue, green and yellow. F) Bar plot of the gene count of NFKB1 (p50) and RELA (p65) in NECs, A549-TECs, H1755-TECs and H1975-TECs. RMFI: Relative mean of fluorescence. Data are mean ± SEM, n > 5, *p < 0.05, Wilcoxon test. G) Representative picture (n=2) of immunoblotting for p65 and its phosphorylated Ser 536 form in NECs and NSCLC-TECs.


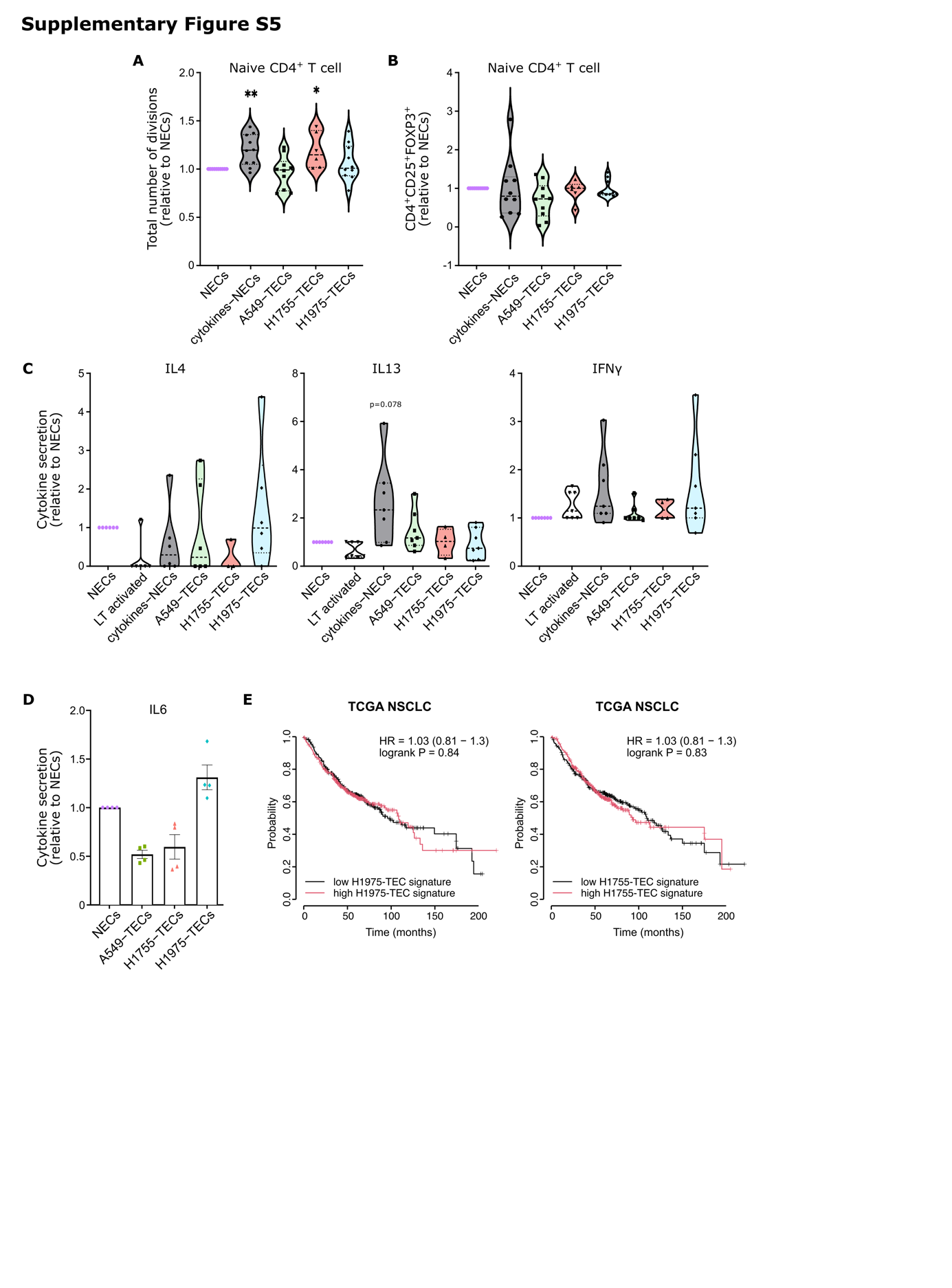


**Supplementary Figure S5** **– NSCLC-TECs have limited impact on naive CD4^+^ T cell polarization.** A) Violin plot of the total number of divisions of naive CD4^+^ T lymphocytes after coculture with NECs treated with TNFα/IL-1ß pro-inflammatory cytokines (cytokines-NECs), A549-TECs, H1755-TECs and H1975-TECs compared to NECs. B) Violin plot of the number of naive CD4^+^ CD25^+^FoxP3^+^ cells after coculture with cytokines-NECs, A549-TECs, H1755-TECs and H1975-TECs compared to NECs. C) Violin plot of IL4, IL13 and IFNγ secretion after naive CD4^+^ T lymphocytes coculture with cytokines-NECs, A549-TECs, H1755-TECs and H1975-TECs compared to NECs, and in activated lymphocytes using LEGENDplex Human T Helper Cytokine Panels. D) Bar plot of IL6 secretion after naive CD4^+^ T lymphocytes coculture with A549-TECs, H1755-TECs and H1975-TECs compared to NECs. E) Survival probability of NSCLC patients from the TCGA database regarding a low or high H1755- or H1975-TEC signature. Data are mean ± SEM, n > 4, *p < 0.05, **p < 0.01, Wilcoxon test compared to NECs.


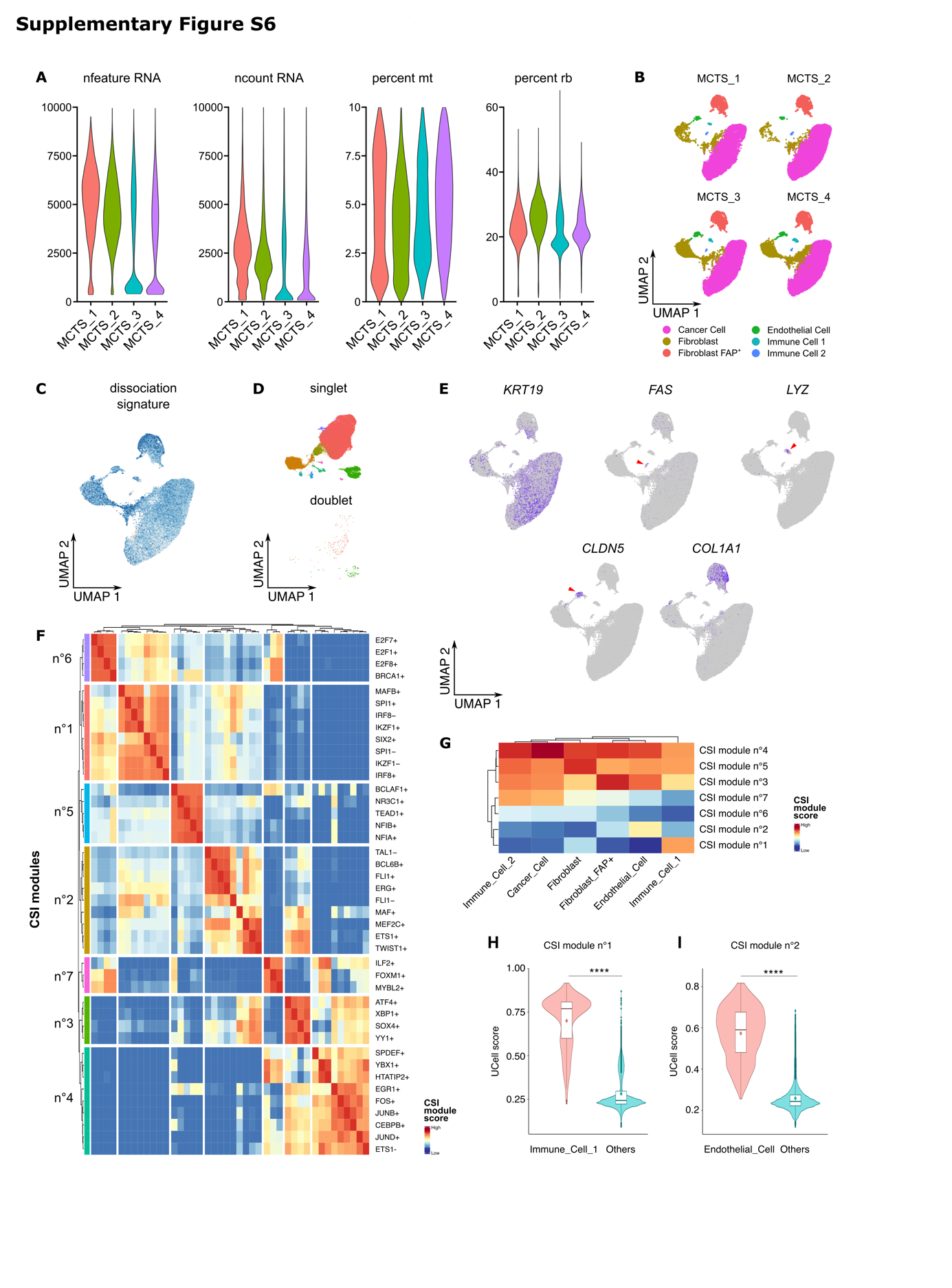


**Supplementary Figure S6** **– Quality metrics of MCTS scRNA-seq and SCENIC analysis.** A) Violin plot of nfeature RNA, ncount RNA, mitochondrial (mt) or ribosomal (rb) percentages in the 4 MCTS donors. B) UMAP representing the diversity of the cell types identified in the 4 different donors. C) UMAP of the dissociation signature score. No score enrichment was identified in a specific cluster. D) UMAP of the singlet and doublet of MCTS. E) UMAP of the expression of key marker genes in MCTS. The expression of *KRT19, FAS, LYZ*, *CLDN5* and *COL1A1* is represented. F) Heatmap of the CSI matrix and hierarchical clustering of regulons in modules. +: activator, -: repressor. G) Heatmap of the mean Ucell score of modules on regulons AUC in the different cell subtypes identified in MCTS. H-I) Violin plot showing the enrichment of co-expression H) module 1 in the Immune Cell 1 cluster, and I) module 2 in the Endothelial Cell cluster.


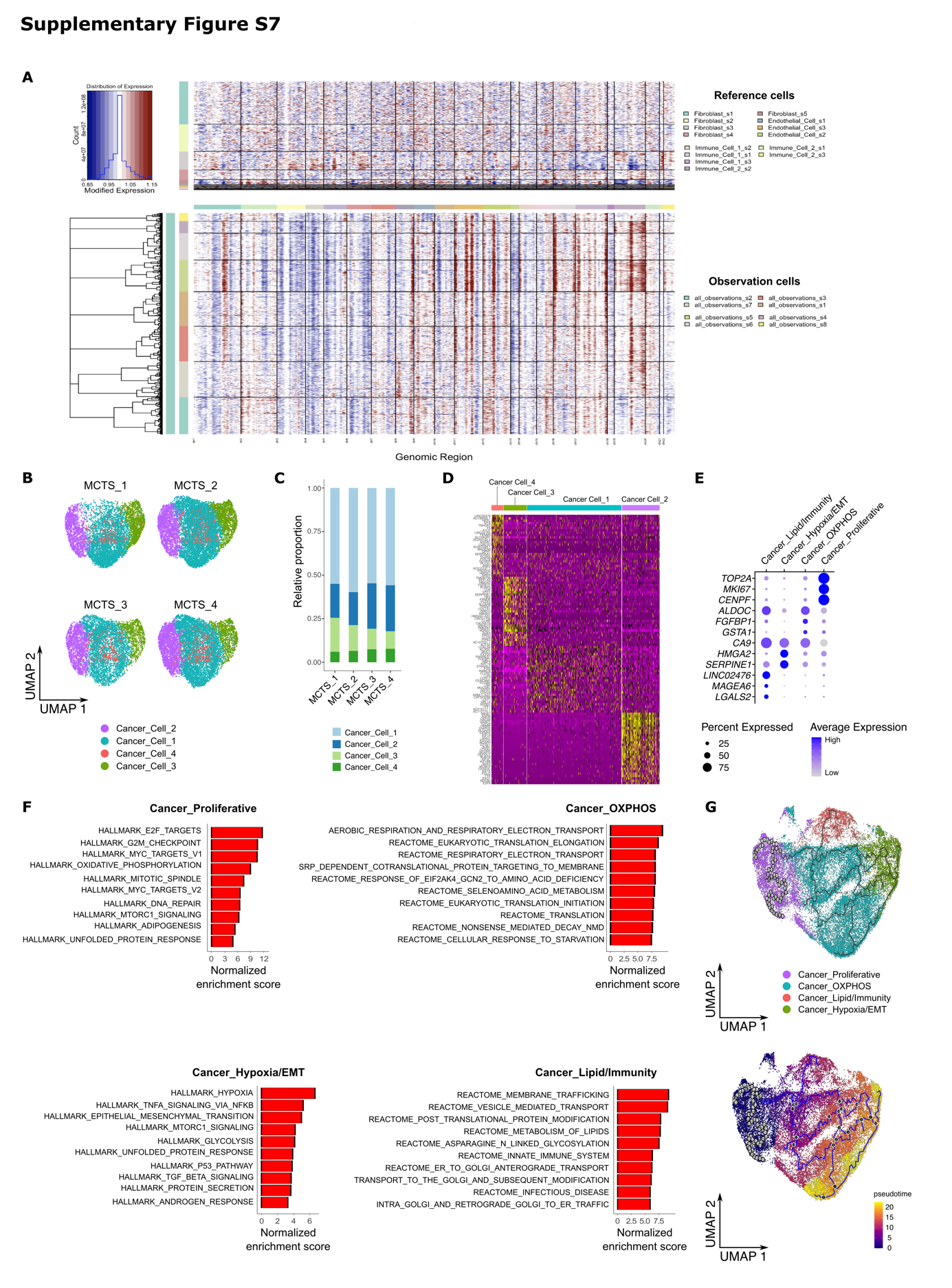


**Supplementary Figure S7** **– Tumor cell heterogeneity within MCTS.** A) Heatmap of the copy number variation (CNV) obtained by inferCNV on the different cell types identified regarding the genomic region. Reference cells selected were all non-tumor cells. B) UMAP and C) relative proportions of the 4 sub-clusters identified in the cancer cells clusters across the 4 different MCTS donors. D) Heatmap of the most significant top-50 deregulated genes in the cancer cells sub-clusters. E) Dot plot of the expression level of the key genes in the 4 cancer cells sub-clusters. The 4 sub-clusters are Cancer_Proliferative, Cancer_OXPHOS, Cancer_Hypoxia/EMT, Cancer_Lipid/Immunity. F) Gene set enrichment analysis of the upregulated pathways in the different cancer cell sub-clusters. G) UMAP of the pseudotime trajectory of the cancer cells sub-clusters. Proliferative cancer cells were selected as starting point.


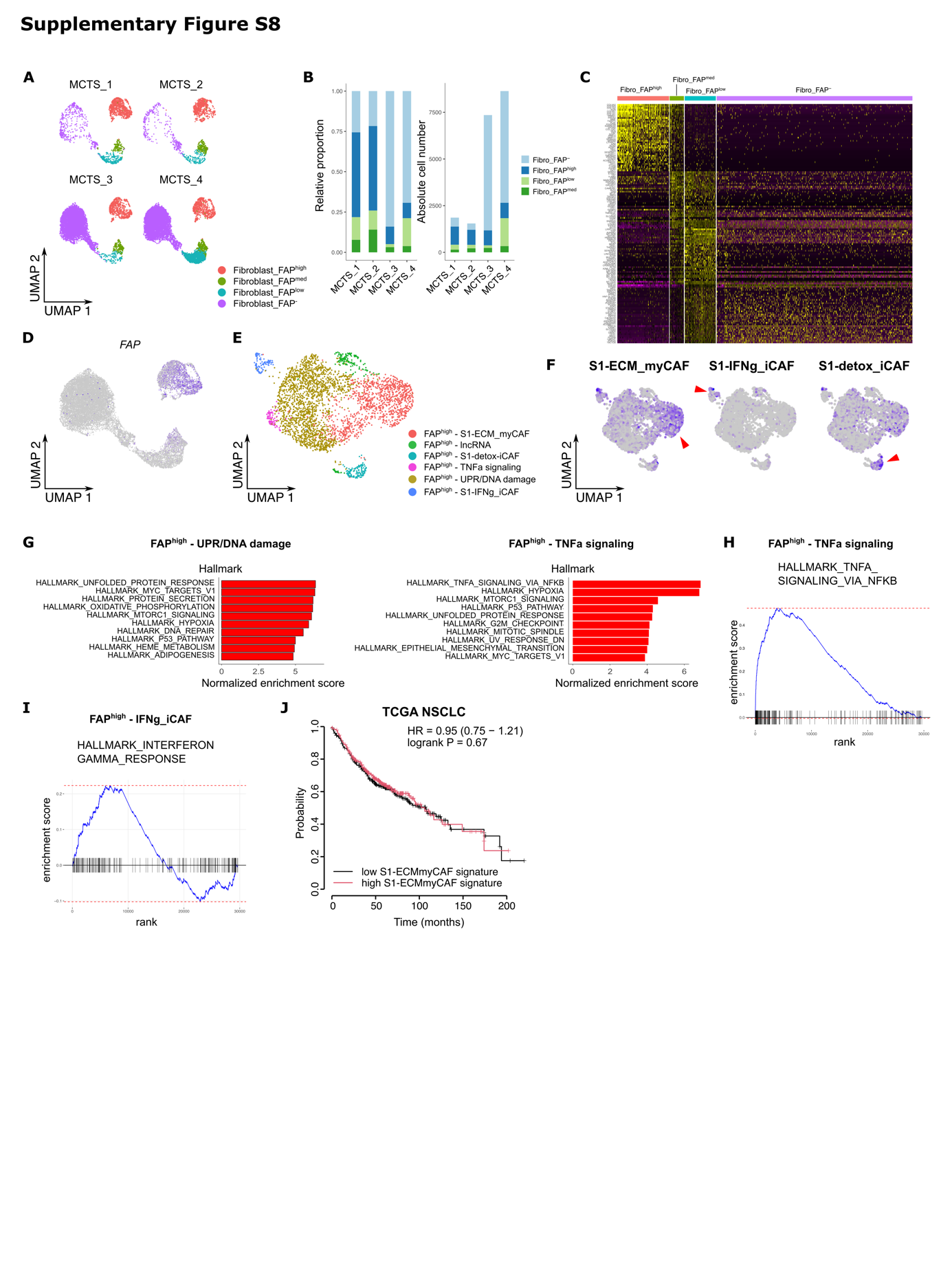


**Supplementary Figure S8 – Fibroblast heterogeneity within MCTS.** A) UMAP and B) relative proportion of the fibroblast clusters across the 4 different MCTS donors. C) Heatmap of the most significant top-75 deregulated genes in the fibroblasts sub-clusters. D) UMAP representation of FAP expression in MCTS. E) UMAP of the sub-clusters identified in FAP^high^ fibroblasts. The sub-clusters are FAP^high^-S1-myCAF, FAP^high^-lncRNA, FAP^high^-S1-detox-iCAF, FAP^high^-TNFa signaling, FAP^high^-UPR/DNA damage, FAP^high^-IFNg_iCAF. F) UMAP representation of the S1 CAF signature identified: S1-ECM_myCAF, S1-IFNg_iCAF and S1-detox_iCAF. G) Gene set enrichment analysis of the upregulated pathways in the sub-clusters FAP^high^-UPR/DNA damage and FAP^high^-TNFa signaling. H) Graph of the enrichment score of HALLMARK_TNFA_SIGNALING_VIA_NFKB in FAP^high^-TNFa signaling sub-clusters, and I) HALLMARK_INTERFERON_GAMMA_ RESPONSE in FAP^high^-IFNg-iCAF sub-clusters. J) Survival probability of NSCLC patient using TCGA dataset depending on the high or low S1-ECM_myCAF signature.

**
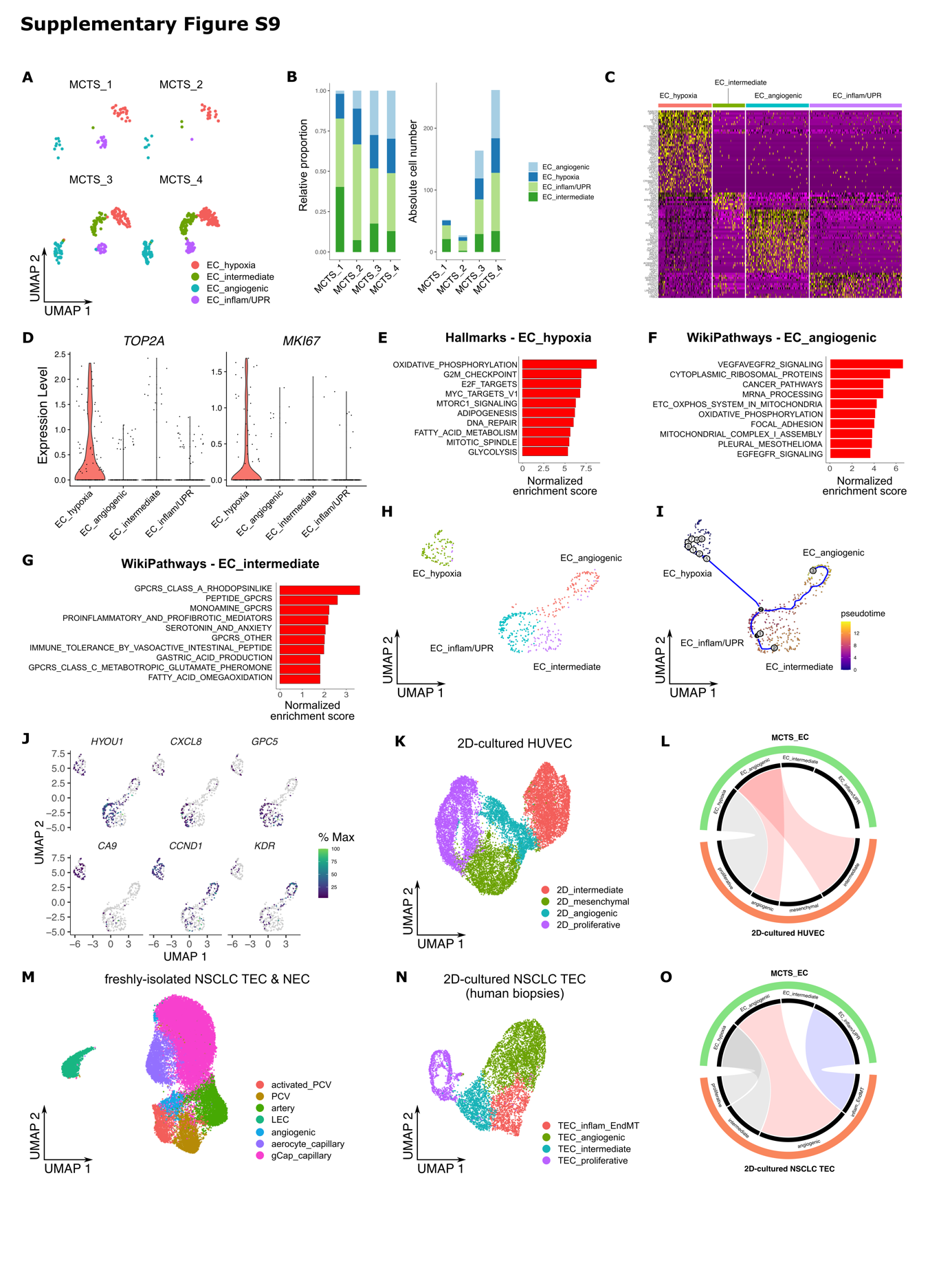
**

**Supplementary Figure S9 – EC heterogeneity within MCTS and comparison to other EC models.** A) UMAP and B) relative proportion of the EC sub-clusters across the 4 different MCTS donors. C) Heatmap of the most significant top-50 deregulated genes in the endothelial sub-clusters. D) Violin plot of the expression level of *TOP2A* and *MKI67* in the different endothelial cells sub-clusters. Note the enrichment in the EC_hypoxia cluster. Gene set enrichment analysis of the upregulated pathways in the sub-cluster E) EC_hypoxia, F) EC_angiogenic and G) EC_intermediate. H-I) Pseudotime trajectory analysis in the EC cluster. EC_hypoxia with proliferative feature were selected as starting point. I) UMAP of the trajectory pseudotime of the EC sub-clusters. J) UMAP of the gene expression of *HYOU1, CXCL8, GPC5*, *CA9, CCND1* and *KDR* in EC type in pseudotime. K) UMAP of the sub-clusters identified in 2D-cultured HUVECs. L) Circleplots showing similarities between MCTS-ECs and 2D-cultured HUVECs. M) UMAP of the sub-clusters identified in freshly isolated human NSCLC-TECs and NECs. N) UMAP of the sub-clusters identified in 2D cultured NSCLC TECs isolated from human biopsies. O) Circleplots showing similarities between MCTS-ECs and 2D-cultured NSCLC-TECs.

**
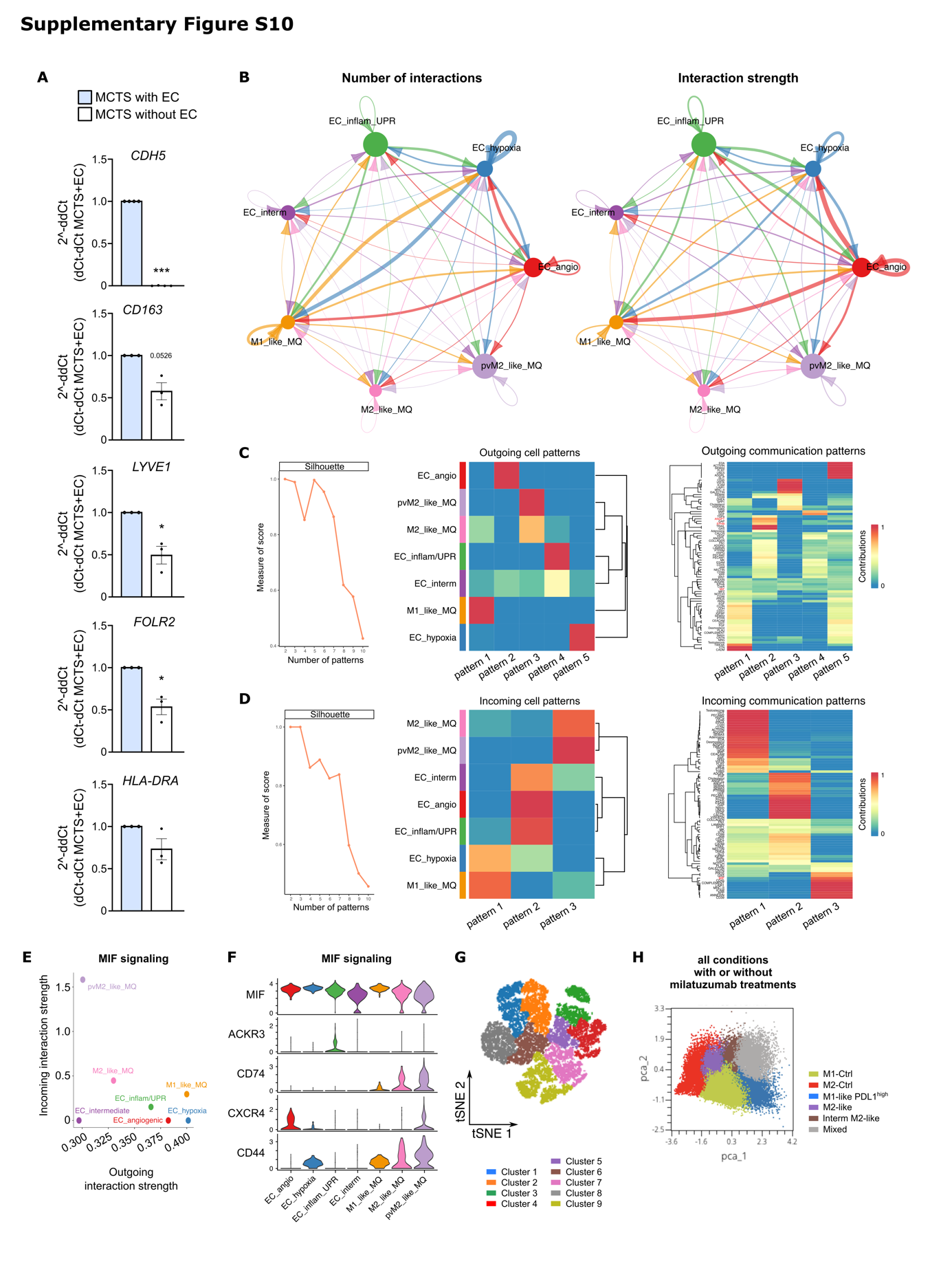
**

**Supplementary Figure S10 – Predicted cell-cell interactions between ECs and macrophages within MCTS.** A) RT-qPCR analysis of MCTS formed with and without ECs. Data are mean ± SEM, n > 3, *p < 0.05, ***p < 0.001, Kruskal-Wallis test compared to MCTS with ECs. B) Circleplot showing the number of interaction and the strength of the interaction identified between the different EC sub-clusters and the different macrophages sub-clusters. C-D) Global C) outgoing or D) incoming communication patterns in the endothelial and macrophage sub-clusters. The contribution score is represented by colors going from red (1) to blue (0). E) Cell-chat ligand-receptor predicting incoming and outgoing interactions between endothelial and macrophages sub-clusters in MIF signaling. F) Violin plot of the key marker genes related to MIF signaling pathway expressed in the endothelial and macrophages sub-clusters. G) tSNE representation of the different clusters identified (in the CD31^-^CD45^+^ population) in the AHM tricultures and in the M1-/M2-induced controls, treated or not with the milatuzumab CD74-blocking antibody. H) PCA of the identified clusters in the M1-, M2-controls and AHM triculture conditions upon treatment or not with milatuzumab.
